# Enzyme Activity by Design: An Artificial Rhodium Hydroformylase for Linear Aldehydes

**DOI:** 10.1002/anie.201705753

**Published:** 2017-09-13

**Authors:** Amanda G. Jarvis, Lorenz Obrecht, Peter J. Deuss, Wouter Laan, Emma K. Gibson, Peter P. Wells, Paul C. J. Kamer

**Affiliations:** ^1^ School of Chemistry University of St Andrews North Haugh St Andrews Fife KY16 9ST UK; ^2^ Department of Chemical Engineering (ENTEG) University of Groningen Nijenborgh 4 9747 AG Groningen The Netherlands; ^3^ Department of Chemistry University College London 20 Gordon Street London WC1H 0AJ UK; ^4^ UK Catalysis Hub Research Complex at Harwell Rutherford Appleton Laboratory Harwell Science & Innovation Campus Didcot Oxfordshire OX11 0FA UK; ^5^ School of Chemistry University of Southampton Southampton SO17 1BJ UK; ^6^ Diamond Light Source Harwell Science & Innovation Campus Didcot Oxfordshire OX11 0DE UK; ^7^ Bioinspired Homo- & Heterogeneous Catalysis Leibniz Institute for Catalysis Albert-Einstein-Strasse 29a 18059 Rostock Germany; ^8^ Current address: Phoreon Bioincubator I Gaston Geenslaan 1 3001 Leuven Belgium

**Keywords:** artificial metalloenzymes, catalyst design, hydroformylation, phosphines, rhodium

## Abstract

Artificial metalloenzymes (ArMs) are hybrid catalysts that offer a unique opportunity to combine the superior performance of natural protein structures with the unnatural reactivity of transition‐metal catalytic centers. Therefore, they provide the prospect of highly selective and active catalytic chemical conversions for which natural enzymes are unavailable. Herein, we show how by rationally combining robust site‐specific phosphine bioconjugation methods and a lipid‐binding protein (SCP‐2L), an artificial rhodium hydroformylase was developed that displays remarkable activities and selectivities for the biphasic production of long‐chain linear aldehydes under benign aqueous conditions. Overall, this study demonstrates that judiciously chosen protein‐binding scaffolds can be adapted to obtain metalloenzymes that provide the reactivity of the introduced metal center combined with specifically intended product selectivity.

The development of substrate‐ and product‐specific catalytic processes that operate efficiently at mild reaction temperatures is a major challenge for the synthetic chemistry community.^1^ Enzymes are nature's main catalysts and catalyze numerous chemical transformations, typically under benign conditions. However, many desired chemical reactions are not performed by nature, and therefore, there are no suitable natural enzymes. Artificial metalloenzymes (ArMs) provide a way to bridge that gap between natural and chemical synthesis, providing enzymes for unnatural reactions.^2^ Despite these successes, most ArMs do not meet the rates and performances achieved by natural enzymes.^3^ In addition, the molecular recognition and shape selectivity of proteins have typically not been exploited. The most successful approach to create ArMs has been the use of non‐covalent anchoring strategies utilizing protein scaffolds with strong supramolecular recognition motifs such as avidin.^4^ In these ArMs, the binding site is used to carry the active metal center or bind metal‐containing cofactors, restricting the possible applications of the binding properties of the protein. An alternative approach utilizes site‐selective protein modification methods^5^ to incorporate transition metals into a wide range of protein scaffolds whilst leaving the protein's innate binding capabilities largely intact. Any protein scaffold can be used, allowing the exploitation of the almost unlimited range of highly specific substrate‐binding capabilities of proteins. Therefore, virtually any organometallic non‐natural catalytic reaction can be merged with the sophisticated biological performance of enzymes, and this approach offers significant opportunities for the design of ArMs aiming at high selectivity by shape‐selective product formation. Herein, we demonstrate the potential of such ArMs through the development of artificial rhodium enzymes derived from a protein scaffold that was selected for its apolar substrate‐binding properties. These ArMs enable selective aldehyde formation in the biphasic hydroformylation of long‐chain linear alkenes, a reaction for which no natural enzymes are known, and which is challenged in current industrial applications by the low solubility of the substrates in the aqueous phase.^6^

We have previously reported a method that enables such an approach for the synthesis of ArMs containing metal‐binding phosphine ligands (Scheme 1 A).5a, 7 Rhodium–phosphine complexes are known to be highly active and robust hydroformylation catalysts, and thus our strategy provides the prospect of enzymatic hydroformylation reactions. Rhodium–protein hybrids tested to date in hydroformylation have utilized dative protein–rhodium interactions with limited success.^8^ Although this has led to unprecedented linear selectivities for the hydroformylation of styrene,^9^ the exact nature of the active species, and thus the origin of the selectivity, is still unclear.^10^ Rhodium‐catalyzed hydroformylation is used on a 800 000 ton scale to produce butyraldehyde from propene under biphasic conditions,^11^ allowing for the recovery of the expensive rhodium 3,3′,3′′‐phosphanetriyltris(benzenesulfonic acid) trisodium salt catalyst (Rh/TPPTS). Long‐chain aldehydes are desired by industry as they are important precursors for the production of detergents and plasticizers.^12^ This process is not feasible for long‐chain alkenes (>5 C atoms) owing to their low solubility in water.^6^

**Scheme 1:**
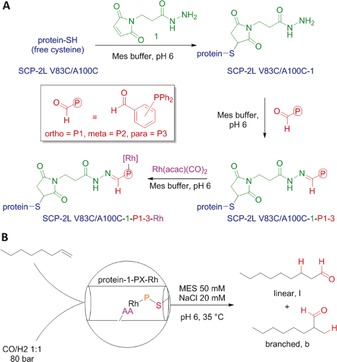
A) Synthesis of the artificial metalloproteins. B) Hydroformylation of 1‐octene. AA=amino acid.

Fatty acid transporter proteins contain apolar tunnels and clefts to bind their hydrophobic cargo. The steroid carrier protein type 2 like (SCP‐2L) domain of the human multifunctional enzyme 2 (MFE‐2) was identified as a suitable linear alkene binding protein scaffold as it is known to bind a variety of linear aliphatic substrates^13^ and can be obtained in high yields.5a Our hypothesis was that the apolar tunnel present in SCP‐2L (Figure 1 B) would be able to facilitate the transport of alkenes to the aqueous environment and orient the starting alkene along the tunnel towards the rhodium center, enabling the shape‐selective production of the desired linear product (Figure 1 A). To introduce the catalytic rhodium–phosphine complexes, two mutants containing unique cysteines at either end of the tunnel were prepared (SCP‐2L V83C and SCP‐2L A100C; Figure 1 B).5a, 14 These two mutants, obtained in excellent yields, showed little structural permutations and similar aliphatic substrate binding capabilities as the wild‐type (WT) protein (see the Supporting Information, Table S3). Both SCP‐2L mutants were successfully modified with aldehyde phosphines **P1**–**P3** through a cysteine modification strategy (Scheme 1 A; for characterization data for SCP‐2L V83C–**1**–(**P1**–**P3**), see Ref. 5a; for SCP‐2L A100C–**1**–(**P1**–**P3**), see the Supporting Information).

**Figure 1:**
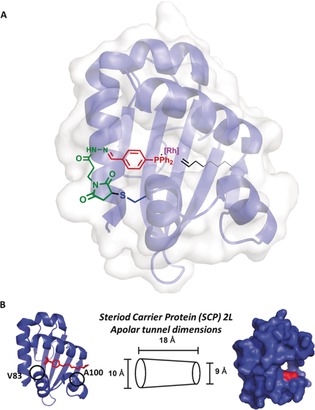
A) The use of the apolar tunnel to introduce regioselectivity into hydroformylation. B) The apolar tunnel in SCP‐2L, showing the position of Triton X‐100 in the tunnel in the original crystal structure (PDB No. 1IKT),^13^ the tunnel dimensions, and the positions of A100 and V83.

The rhodium proteins (SCP‐2L V83C/A100C–**1**–(**P1**–**P3**)–Rh) were obtained by the addition of Rh(acac)(CO)_2_. Other Rh precursors did not selectively bind to the phosphine (see Table S4 for MS and metal loading analysis)_._ Their catalytic activity was investigated in the hydroformylation of 1‐octene at 35 °C and 80 bar synthesis gas (Figure 2, Scheme 1 B, and Table S5). To minimize rhodium leaching and therefore false results from “free Rh” (leading to low selectivity (ca. 55 % linearity) and high turnover numbers (TONs>500; Table 1, entry 2)), a slight excess of protein (2 equiv) was used. Even though these reactions were performed at a relatively low temperature (typical industrial conditions are 125 °C), significant hydroformylation activity was detected over 48 h for several of the rhodium–phosphine ArMs (Figure 2 A). Reactions over time showed that the enzyme was active over the whole 48 h (Table S6). Control reactions with the ArM phosphine selenide or ArM phosphine gold complexes showed that the rhodium was required for the hydroformylation reaction to occur (Table S5).

**Figure 2:**
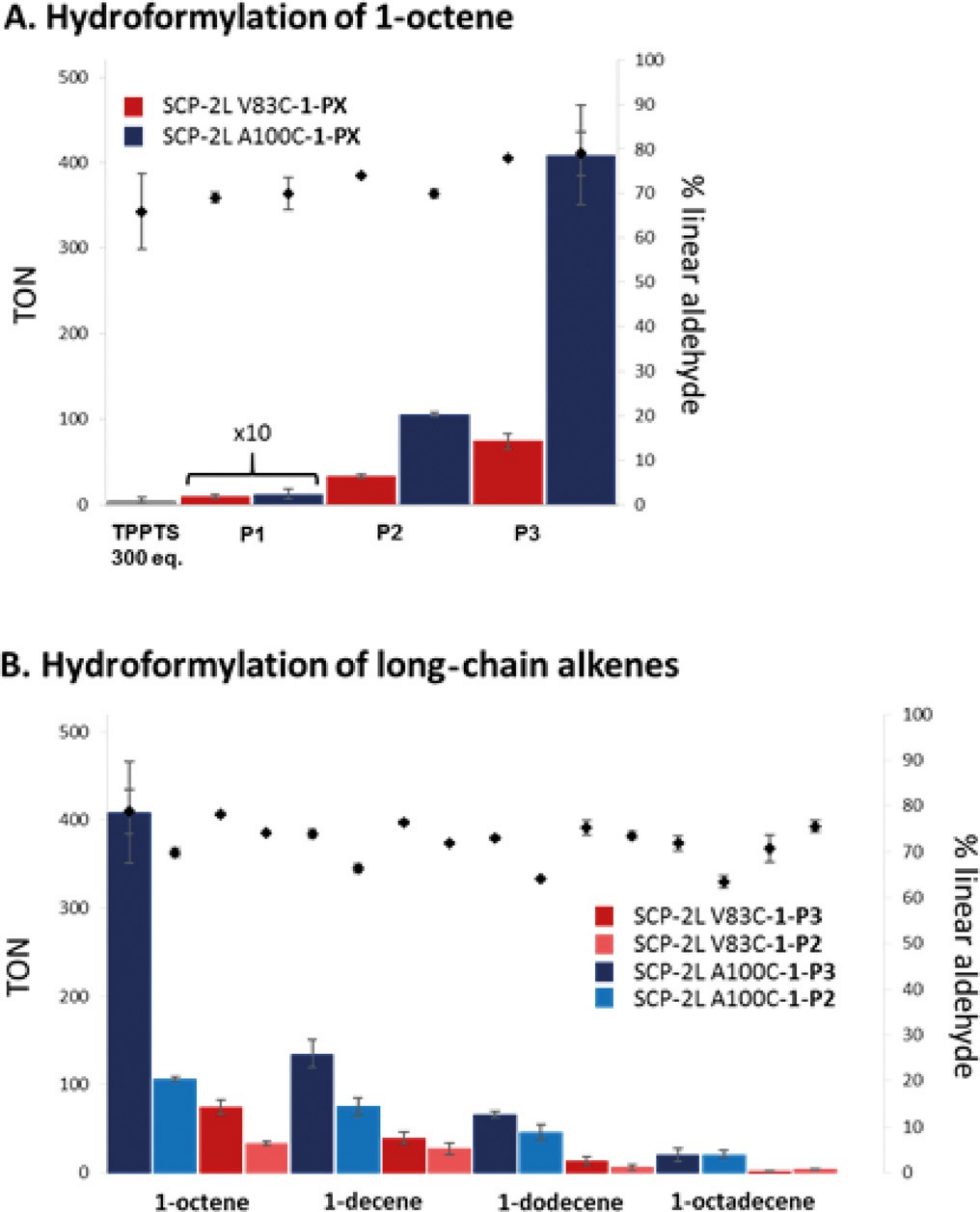
Activity (colored bars) and selectivity (black squares) A) of the catalytic hydroformylation of 1‐octene using different artificial metalloenzymes (the values for **P1** have been magnified) and B) for the hydroformylation of alkenes with different chain lengths. Standard reaction conditions: 80 bar CO/H_2_ (1:1), 35 °C, 625 rpm, 0.5 mL of catalyst solution and 0.5 mL of alkene containing 9 % (v/v) *n*‐heptane and 1 % (v/v) diphenyl ether as internal standards. The Rh concentrations were determined by ICP‐MS and used to calculate the TONs, recorded values between 20 and 100 nmol Rh. Conversions and linear selectivities were obtained by GC analysis with a minimum of three runs. Error bars show the standard deviation. For further details, see Section S4.

**Table 1:** Control reactions for the aqueous hydroformylation of 1‐octene.

Entry	Catalyst		TON			Linear aldehyde [%]	
1	ArM: SCP‐2L A100C–**1**–**P3**–Rh^[a]^		408.7	(57.79)		78.8	(4.86)
2	Rh(acac)(CO)_2_^[b]^		529.7	(53.30)		55.3	(0.67)
3	protein scaffold A100C treated with Rh^[c,d]^		123.5	(38.19)		57.8	(0.07)
4	Rh/TPPTS (1:2)^[e]^		2245	(674)		58.9	(0.41)
5	Rh/TPPTS (1:20)^[e]^		700	(190)		56.5	(0.08)
6	Rh/TPPTS (1:300)^[e]^		5.4	(3.25)		65.9	(8.55)
7	Rh/TPPTS (1:300) and SCP‐2L A100C^[f]^		9.4	(1.01)		60.7	(1.48)
8	Rh/TPPTS (1:300) and WT SCP‐2L^[f]^		5.1	(2.48)		67.0	(0.64)

Standard conditions: 80 bar syngas (1:1), 35 °C, 625 rpm, 0.5 mL of catalyst solution and 0.5 mL of 1‐octene containing 9 % (v/v) *n*‐heptane and 1 % (v/v) diphenyl ether. The Rh concentration was determined by ICP‐MS for entry 1. Conversions and linear selectivities were obtained by GC analysis of a minimum of three runs. Standard deviations given in parentheses. [a] P/Rh=1.5, 23 nmol Rh. [b] 41.25 h, total volume: 0.4 mL 1‐octene, no water, 150 nmol Rh. [c] Treated with Rh and washed in the same manner as the metalloproteins. [d] 10.0 nmol Rh. [e] 30 nmol Rh. [f] Two equivalents of the protein relative to Rh.

The structure of the phosphine cofactor was found to have a large effect on the TON, with the activity increasing by factors of 30 and 70, respectively, as the phosphine moved from the *ortho* (**P1**) to the *meta* position (**P2**) and from the *ortho* (**P1**) to the *para* position (**P3**) for SCP‐2L V83C. The protein scaffold also influenced the reactivity, with a marked improvement in turnover found when using SCP‐2L A100C–**1**–**P3**, achieving TONs of >400, versus 75 for SCP‐2L V83C–**1**–**P3**. The selectivity of the reaction was also found to depend on the combination of protein scaffold and phosphine cofactor applied. It ranged from 69 to 79 % for the linear product (nonanal), matching typical selectivities for double‐phosphine‐ligated rhodium‐catalyzed hydroformylation.^15^ Selectivities of 80 % are rarely seen with monoligated P/Rh systems. Control reactions with **P1**–**P3** or Rh(acac)(CO)(PPh_3_) in neat alkene showed that with monophosphines in the organic phase of our reaction, selectivities of up to 74 % can be achieved (Tables S8 and S9). Catalyst degradation and leaching of the phosphine and rhodium to the organic phase were not responsible for the observed selectivities as only minimal rhodium leaching and degradation were observed (Table S11 and Figures S12 and S13). It should be noted that our ArM system has a low P/Rh ratio, which, when using a benchmark biphasic Rh/TPPTS catalytic system, only gives activities and selectivities that correspond to rhodium leaching into the organic phase (Table 1, entries 2, 4, and 5). At the same concentration as in our ArM reactions, over 300 equivalents of the TPPTS ligand are needed to prevent metal leaching (Table 1, entries 4–6) and obtain high selectivities. The same effect of rhodium leaching was observed when the protein was simply mixed with Rh(acac)(CO)_2_ (Table 1, entry 3). This, alongside the differences between the two protein mutants, shows that it is the hybrid catalysts that are responsible for the hydroformylation results. SCP‐2L A100C–**1**–**P3** gave the best performance overall (79 % nonanal, TON=409; Table 1, entry 1). These conversions and selectivities are remarkable as a benchmark catalyst (Rh/TPPTS) gives negligible conversion approaching the detection limit (TON≈1) when the TPPTS/Rh ratio is optimized to give similar selectivities to the metalloenzyme (TPPTS/Rh=30:1 at ca. 10 times the Rh concentration of the ArM reaction gives 72 % linearity; see Table S7).

Following the successful hydroformylation of 1‐octene, these artificial metalloenzymes were tested in the hydroformylation of 1‐decene, 1‐dodecene, and 1‐octadecene (Figure 2 B).^6^ When TPPTS was used, a tenfold rate decrease was observed on the addition of two carbon atoms to the chain owing to the reduced water solubility of the alkene.^6^ Using the ArM, a less than fourfold decrease in activity was observed upon going from 1‐octene to 1‐decene, and an only tenfold decrease when going to octadecene. Under selectivity‐optimized conditions (high ligand concentrations to give adequate linear selectivity while preventing Rh leaching), Rh/TPPTS displayed no significant activity (Figure 2 A, Table 1, entry 6, and Table S7). Control experiments using the Rh/TPPTS system in the presence of the protein scaffold showed no significant difference in turnover for 1‐octene (Table 1, entry 6 vs. 7 and 8), providing evidence that the increase in activity for the ArMs cannot solely be explained by the protein acting as a phase‐transfer reagent. We therefore attribute the higher‐than‐expected activity to the presence of the lipid‐binding tunnel in the protein scaffold in direct proximity of the Rh center.

Overall, the selectivities for the linear hydroformylation products were remarkably high for monophosphine‐ligated rhodium in water, indicating that the protein scaffold counterbalances the lack of phosphine ligands. In addition, both the phosphine cofactor and the protein mutant affect the activity of the reaction. To better understand the observed selectivity of our hydroformylase, we investigated the local environment of the Rh center in the protein scaffold. Both the X‐ray absorption near‐edge structure (XANES) and extended X‐ray absorption fine structure (EXAFS) of SCP‐2L A100C–**1**–**P3**–Rh at the Rh K edge were assessed. Comparing the XANES of SCP‐2L A100C–**1**–**P3**–Rh to model Rh complexes (Figure S10) suggested the loss of carbonyl functionalities from the Rh(acac)(CO)_2_ precursor. This was further supported by the lack of CO stretches in the IR spectrum and fitting the EXAFS data of SCP‐2L A100C–**1**–**P3**–Rh (Table S1).

The fitting model applied used characteristic scattering paths from both acac and PPh_2_Ar ligands, with a derived Rh–P coordination number of two. EXAFS is unable to distinguish between scattering neighbors of like atomic number, especially where *Z*=±1. The prospect of two phosphorus atoms coordinated to the rhodium center appeared unlikely owing to the steric congestion resulting from placing two protein scaffolds around the metal. Thus we postulated the possibility of one of the observed Rh–P neighbors arising from a Rh–S interaction (Figure 3 A); the protein scaffold contains functionalized sulfur in the form of methionine, at the N‐terminus and on the flexible α‐helices, and the introduced cysteine (Figure 3 B).

**Figure 3:**
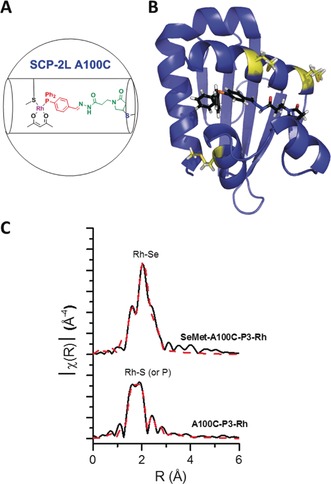
A) Cartoon of the hypothesized metal environment. B) Model of A100C (Swiss model) with **1**–**P3** docked using gold to illustrate the reach of this added cofactor. Methionine residues within range are highlighted in yellow. C) The k3‐weighted Rh K edge EXAFS data (black lines) and associated fit (dashed red line) for A100C–**1**–**P3**–Rh and SeMet‐A100C–**1**–**P3**–Rh.

To probe the possibility of a nearby methionine coordinating to the Rh center, the selenomethionine derivatives of the protein scaffold were expressed and purified. Rhodium complexation of the phosphine‐modified SeMet protein with Rh(acac)(CO)_2_ gave similar results as for SCP‐2L A100C–**1**–**P3**, showing the RhCO adducts in the mass spectra, and also resulted in a small shift in the ^77^Se NMR spectrum upon Rh addition (Figure S11). There was a profound change in the Rh K edge EXAFS data of SeMet‐A100C–**1**–**P3**–Rh compared to SCP‐2L A100C–**1**–**P3**–Rh (Figure 3 C). The differences observed can be rationalized by coordination to a neighbor of higher atomic number with a larger backscattering amplitude; between the analogous systems, Rh–S interactions have been replaced by Rh–Se interactions. The EXAFS analysis supports our hypothesis that there is monophosphine coordination, coupled with further interaction with sulfur from a methionine residue. Moreover, through observation of this Rh–S/Se interaction, we have direct evidence for the coordination of the protein scaffold to the Rh center.

We were intrigued if the methionine coordination would have an effect on the catalytic performance of the ArM, or if the methionine would just decoordinate under the reaction conditions to give the same active catalyst, and thus the same catalytic results in all cases. To probe this further, four mutants of SCP‐2L A100C were prepared in which each Met residue was replaced by alanine, and the proteins were then modified as above. The results of the initial catalytic experiments are ambiguous; the activity of SCP‐2L A100C M105A–**1**–**P3** was on average higher than that of A100C or the other mutants (TON=112(±33) and TON=60–86 for the others, see Table S10), which could indicate that M105 is indeed involved in rhodium coordination. However, this can also be due to other factors, such as structural changes or decreased protein stability. More detailed studies are required before firm conclusions can be drawn on the role of the methionine residues in the hydroformylation by these ArMs.

In summary, we have shown that rhodium phosphine modified SCP‐2Ls are linear‐selective catalysts in the hydroformylation of long‐chain alkenes. SCP‐2L A100C–**1**–**P3**–Rh showed a rate enhancement of at least 10^3^ compared to the traditional Rh/TPPTS system in the biphasic hydroformylation of 1‐octene and 1‐decene. This demonstrates that a protein chosen for its specific binding properties can be converted into an enzyme in which these properties are used to transmit product selectivity. Combining this technology with the recent advances in chemical biology will allow us to rapidly engineer highly selective catalysts that operate under benign conditions. Moving forward, we believe that this approach has the potential to be used for a whole range of reactions that traditionally use phosphines as ligands and to convert these into biocatalytic processes. In the long term, as chemogenetic optimization is used to improve activity, this could open the door to a new era of biocatalytic chemical production.

## Conflict of interest

The authors declare no conflict of interest.

## Supporting information

As a service to our authors and readers, this journal provides supporting information supplied by the authors. Such materials are peer reviewed and may be re‐organized for online delivery, but are not copy‐edited or typeset. Technical support issues arising from supporting information (other than missing files) should be addressed to the authors.

Supplementary

## References

[1] Contemporary Catalysis: Science, Technology, and Applications (Eds.: P. C. J. Kamer, D. Vogt, J. Thybaut), RSC, Cambridge, 2017.

[2a] J. C. Lewis , ACS Catal. 2013, 3, 2954–2975;

[2b] F. Rosati , G. Roelfes , ChemCatChem 2010, 2, 916–927;

[2c] A. Chatterjee , H. Mallin , J. Klehr , J. Vallapurackal , A. D. Finke , L. Vera , M. Marsh , T. R. Ward , Chem. Sci. 2016, 7, 673–677; 10.1039/c5sc03116h PMC5953008 29896353

[2d] P. Srivastava , H. Yang , K. Ellis-Guardiola , J. C. Lewis , Nat. Commun. 2015, 6, 7789; 26206238 10.1038/ncomms8789 PMC4525152

[2e] Y. Lu , N. Yeung , N. Sieracki , N. M. Marshall , Nature 2009, 460, 855–862; 19675646 10.1038/nature08304 PMC2770889

[2f] O. Pàmies , M. Diéguez , J.-E. Bäckvall , Adv. Synth. Catal. 2015, 357, 1567–1586.

[3a] T. Heinisch , T. R. Ward , Acc. Chem. Res. 2016, 49, 1711–1721; 27529561 10.1021/acs.accounts.6b00235

[3b] F. Nastri , M. Chino , O. Maglio , A. Bhagi-Damodaran , Y. Lu , A. Lombardi , Chem. Soc. Rev. 2016, 45, 5020–5054. 27341693 10.1039/c5cs00923e PMC5021598

[4a] T. R. Ward , Acc. Chem. Res. 2011, 44, 47–57; 20949947 10.1021/ar100099u

[4b] H. M. Key , P. Dydio , D. S. Clark , J. F. Hartwig , Nature 2016, 534, 534–537. 27296224 10.1038/nature17968 PMC11723505

[5a] P. J. Deuss , G. Popa , C. H. Botting , W. Laan , P. C. J. Kamer , Angew. Chem. Int. Ed. 2010, 49, 5315–5317; 10.1002/anie.201002174 20572235

[5b] Bioconjugate Techniques, 2nd ed. (Ed.: G. T. Hermanson), Acadamic Press, San Diego, 2008.

[6] H. Bahrmann , S. Bogdanovic , P. W. N. M. van Leeuwen in Aqueous Phase Organometallic Catalysis, 2nd ed. (Eds.: B. Cornils, W. Herrmann), Wiley-VCH, Weinheim, 2004, pp. 391–409.

[7a] R. den Heeten , B. K. Muñoz , G. Popa , W. Laan , P. C. J. Kamer , Dalton Trans. 2010, 39, 8477–8483; 20603670 10.1039/c0dt00239a

[7b] W. Laan , B. K. Muñoz , R. den Heeten , P. C. J. Kamer , ChemBioChem 2010, 11, 1236–1239. 20432427 10.1002/cbic.201000159

[8a] M. Marchetti , G. Mangano , S. Pagnelli , C. Botteghi , Tetrahedron Lett. 2000, 41, 3717–3720;

[8b] C. Bertucci , C. Botteghi , D. Giunta , M. Marchetti , S. Paganelli , Adv. Synth. Catal. 2002, 344, 556–562.

[9] Q. Jing , R. J. Kazlauskas , ChemCatChem 2010, 2, 953–957.

[10] H. M. Key , D. S. Clark , J. F. Hartwig , J. Am. Chem. Soc. 2015, 137, 8261–8268. 26020584 10.1021/jacs.5b04431 PMC11620536

[11] E. Wiebus , B. Cornils in Catalyst Separation, Recovery and Recycling. Chemistry and Process Design (Eds.: D. J. Cole-Hamilton, R. P. Tooze), Springer, Amsterdam, 2006, pp. 105.

[12] C. D. Frohning , C. W. Kohlpaintner , H.-W. Bohene in Applied Homogeneous Catalysis with Organometallic Compounds, Vol. 1, 2nd ed. (Eds.: B. Cornils, W. A. Herrmann), Wiley-VCH, Weinheim, 2002 pp. 29–103.

[13] A. M. Haapalainen et al., J. Mol. Biol. 2001, 313, 1127–1138. 11700068 10.1006/jmbi.2001.5084

[14] P. J. Deuss , G. Popa , A. M. Z. Slawin , W. Lann , P. C. J. Kamer , ChemCatChem 2013, 5, 1184–1191.

[15] Rhodium Catalysed Hydroformylation (Eds.: P. W. N. M. Van Leeuwen, C. Claver), Kluwer Academic Publishers, New York, 2002.

