# Supplementary material for: Enzyme Activity by Design: An Artificial Rhodium Hydroformylase for Linear Aldehydes
Source: Angew Chem Int Ed Engl. 2017 Sep 13;56(44):13596–600. doi: 10.1002/anie.201705753 (PMC5659135; doi:10.1002/anie.201705753)
Supplement: Supplementary file 1 — Supplementary [file ANIE-56-13596-s001.pdf]

Angewandte Chemie
Eine Zeitschrift der Gesellschaft Deutscher Chemiker

Supporting Information

Enzyme Activity by Design: An Artificial Rhodium Hydroformylase for Linear Aldehydes

Amanda G. Jarvis, Lorenz Obrecht, Peter J. Deuss, Wouter Laan, Emma K. Gibson, Peter P. Wells, and Paul C. J. Kamer**

anie_201705753_sm_miscellaneous_information.pdf

Table of Contents

Contents

Table of Contents 2
1. Protein expression and purification 3
   SCP-2L V83C: 3
   SCP-2L A100C: 3
   Seleneomethionine protein scaffolds: 5
   Alanine mutants: 6
2. Protein Modification 6
3. Characterisation of the modified proteins 8
   3.i Mass spectrometry on intact proteins and modified proteins: 8
   3.ii. CD: 10
   3.iii. XAFS: 11
   3.iv. NMR: 14
   3.v. Infra-red studies: 15
   3.vi. Protein binding studies: 15
   3.vii. Alternate Rh precursors: 15
4. Hydroformylation 16
   4.i. General hydroformylation procedure: 16
   4.ii. Hydroformylation using phosphine ligands: 17
   4.iii. Hydroformylation of 1-octene with alanine mutants 19
   4.iv. Gas chromatography: 19
   4.v. ICP-MS: 20
   Metal leaching experiments: 20
   4.vi. Characterisation of protein after catalytic reaction: 21
5. Rate calculations 22
References 23
Author Contributions 24

1. Protein expression and purification

SCP-2L V83C:

Protein expression and purification was performed as described in literature.[1]

SCP-2L A100C:

Site directed mutagenesis – the A100C mutation was introduced using the Quick-Change Site-Directed Mutagenesis Kit (Stratagene) with pEHISTEV::dΔhΔSCP-2L as the template. The primers used for the mutagenesis are shown below. The constructs were verified by sequencing by the Sequencing Service, School of Life Sciences, University of Dundee, Dundee.

Primer	Mutation acquired	Sequence
**Primer 1**	A100C GCC/TGC	5' AGTGGCAGGCTGAAG**TGC**AGAGGGAACATCATG 3'
**Primer 2**		5' CATGATGTTCCCTCT**GCA**CTTCAGCCTGCCACT 3'

Sequence of His-tagged SCP-2L A100C:
MSYYHHHHHHDYDIPTTENLYFQGAMEGGKLQSTFVFEEIGRRLKDIGPEVVKKVN
AVFEWHITKGGNIGAKWTIDLKSGSGKVYQGPAKGAADTTIILSDEDF
MEVVLGKLDPQKAFFSGRLKCRGNIMLSQKLQMILKDYAKL

Protein expression and purification was carried out as described for V83C. The procedure is repeated below for clarity:

SCP-2L A100C was prepared by transforming the pEHISTEV::dΔhΔSCP-2L A100C plasmid into competent *E. coli* Rosetta(DE3) cells. A single transformed colony was inoculated into 10 mL PB medium (Production Broth medium; containing 20 g/L tryptone, 10 g/L yeast extract, 5 g/L dextrose, 5 g/L NaCl, 8.7 g/L K_2HPO_4, pH 7.0) with 50 µg/mL kanamycin and 34 µg/mL chloramphenicol (in ethanol). This was used to inoculate 100 mL PB media (50 µg/mL kanamycin and 34 µg/mL chloramphenicol) and agitated at 37 °C, 200 rpm overnight (16-18 h). This starter culture (10 mL) was used to inoculate 0.5 litre of PB medium (2 litres in total). The cells were allowed to grow at 37 °C to an OD_{600} of 0.6 (about 2 h) after which the temperature was lowered to 16 °C. Isopropyl-1-thio-β-D-galactopyranoside (IPTG) was added after one hour to a final concentration of 0.2 mM from a 0.2 M stock solution to initiate the expression of the recombinant protein. The culture was left overnight at 16 °C (16-18 h). The cells were harvested by centrifugation (20 min, 7500 g), washed with 250 mL of buffer solution (16 mM kPi (potassium phosphate) 120 mM NaCl, pH 7.4) and centrifuged again (20 min 7500 g). The pellet from the 2 litre cell culture was resuspended at 4 °C in 100 ml of buffer solution (50 mM Tris·HCl, 20 mM imidazole, 150 mM NaCl, 0.5 mM benzamidine, pH 8) and frozen at -80 °C.

After defrosting, 20 mg of lysozyme, 1 mg of DNase and 1 mL of 1 M of $MgCl_2$ was added to the suspension. The suspension was left for 1 hour at 4 °C and was sonicated in portions of 10 mL (Hielscher UP200S ultrasonic processor, 0.5 s pulses 90% power) with 0.5 second pulses for 1 minute. The cell-extract, obtained after centrifugation (50 min, 30000 g) was filtered (0.22 µm PES Millex Filter unit) and applied to a nickel column (5 mL HisTrap FF) equilibrated with 30 mM Tris·HCl, 20 mM imidazole, 150 mM NaCl, pH 8 (wash buffer) at flow rates up to 5 mL/min. The column was washed with 5 column volumes of wash buffer, 5 column volumes of high salt buffer (wash buffer containing 1 M NaCl) and another 5 column volumes of wash

buffer. The protein was obtained by eluting with 6 column volumes elution buffer (wash buffer containing 330 mM imidazole) into an equal volume of wash buffer to prevent precipitation of the protein and the resulting solution was dialysed against 5 litre buffer solution (30 mM Tris·HCl, 10 mM imidazole, 150 mM NaCl, pH 8 at 4°C). If precipitate had formed the resulting mixture was centrifuged (30000 g, 50 min). 0.014 equivalents of TEV-protease[2] and final concentrations of 1 mM DTT and 0.5 mM EDTA were added to resulting His-tagged SCP-2L A100C solution. This mixture was left overnight at room temperature.

If precipitate had formed the mixture was again centrifuged (50 min, 30000 g) and the pellet was discarded. The supernatant was then filtered (0.22 µm PES Millex Filter unit) and used for nickel affinity chromatography on a nickel column (5 mL HisTrap FF) equilibrated with wash buffer. The flow-through containing pure SCP-2L A100C was collected, concentrated and the buffer was exchanged to the storage and coupling buffer (20 mM MES, 50 mM NaCl, pH 6) using a centrifugal concentrator. Three consecutive rounds of buffer exchange were performed to remove all the DTT. Buffer exchange by centrifugal concentrator was found to be more effective than dialysis. Yields up to 75 mg L^{-1} culture, with a purity of more than >99% according to SDS-PAGE (Nupage™ 4-12% Bis-Tris gel), were obtained.

Ladder: Mark 12™, Novex
2: Histagged SCP-2L A100C (showing disulfide present). Expected mass: 16.37 kDa
3: SCP-2L A100C (after TEV). Expected mass: 13.40 kDa

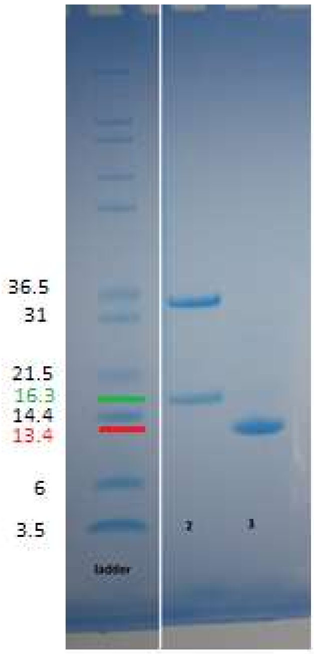

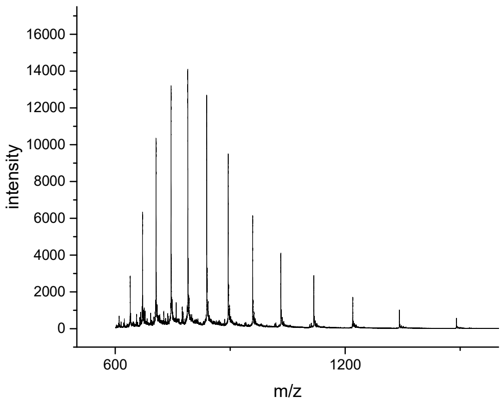

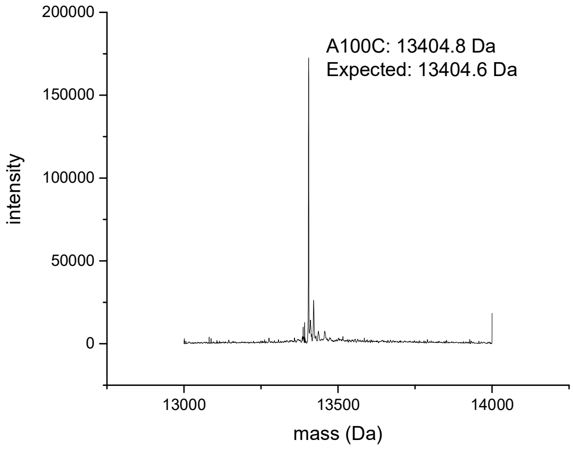

Figure S1: Raw mass spectrum of SCP-2L A100C (top) and the deconvoluted mass-spectrum of SCP-2L A100C (bottom) obtained by LC-MS (ES+). Pictures created using Origin.

Seleneomethionine protein scaffolds:

Selenomethionine (SeMet) derivatised SCP-2L A100C was prepared by transforming the pEHISTEV::dΔhΔSCP-2L A100C plasmid into competent *E. coli* B834 (DE3) cells. A single transformed colony was inoculated into 10 mL LB medium (Lysogeny broth medium contains: 10 g/L tryptone, 5 g/L yeast extract, 5 g/L NaCl, pH 7.0) and agitated at 37 °C, 200 rpm for 8 h, this was then used to inoculate 100 mL LB medium (containing 50 µg/mL kanamycin) and agitated at 37 °C, 200 rpm overnight (16 h). The starter culture was pelleted and washed twice with minimal media to remove the LB medium (and thus methionine), and resuspended in minimal media. The SeMet media was prepared by dissolving 5.1 g of nutrient mix (SelenoMet™ Nutrient Mix, Molecular Dimensions) in ddH_2O (50 mL) and sterile filtering into 1 L base media (NH_4Cl 1 g/L, KH_2PO_4 3 g/L and $(Na_2HPO_4)_7H_2O$ 6 g/L containing 50 µg/mL kanamycin). The SeMet media (4 x 750 mL) was inoculated with 10 mL of the resuspended cells and incubated at 37 °C, 200 rpm for 20 min. L-Seleneomethionine was then added to the cell culture to a final concentration of 60 µg/mL. Incubation at 37 °C continued until the OD_{600} reached 0.6 (about 2 h), and the cells were induced with 0.2 M IPTG (1 mL per flask). The cells were allowed to grow overnight (16-20 h) at 16 °C with agitation (200 rpm). The cells were harvested by centrifugation (7500 g, 25 min, 4 °C), washed with 500 mL of buffer solution (16 mM kPi (potassium phosphate) 120 mM NaCl pH 7.4) and centrifuged again (7500 g, 25 min, 4 °C). The pellet was resuspended in 2x 45 mL of buffer solution (50 mM Tris·HCl, 20 mM imidazole, 150 mM NaCl, 0.5 mM benzamidine, pH 8) and frozen at -80 °C.

After defrosting, 15 mg of lysozyme, 1 mg of DNase and 1 mL of 1 M of $MgCl_2$ was added to each portion of the suspension. The suspension was left for 1 hour at 4 °C, then each 45 mL portion was lysed by sonicated on ice (5 x 60 sec, 0.5 s pulses of 75% power, shaking between each repeat). The cell-extract, obtained after centrifugation (35000 g, 45 min, 4 °C) was filtered, and purified using Ni^{II}-affinity chromatography (5 mL HisTrap FF) as described for SCP-2L A100C. Yield obtained: 25 mgL^{-1} media.

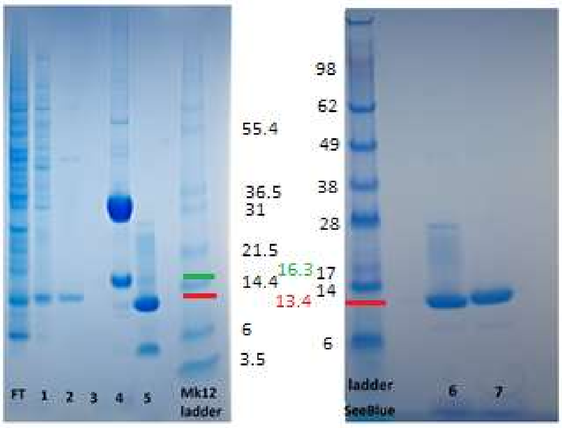

SDS page gel (Nupage™ 4-12% Bis-Tris gel):
FT: Flow through from column
1: 1st wash
2: HS wash
3: 2nd wash
4: Elution
5: Monitoring TEV reaction (before final purification by Ni column)
6: Pure SCP-2L A100C (13.4 kDa)
7: SCP-2L A100C-**1** (13.58 kDa)
Ladders: Mark 12™ unstained and SeeBlue® Plus 2 prestained from Novex.

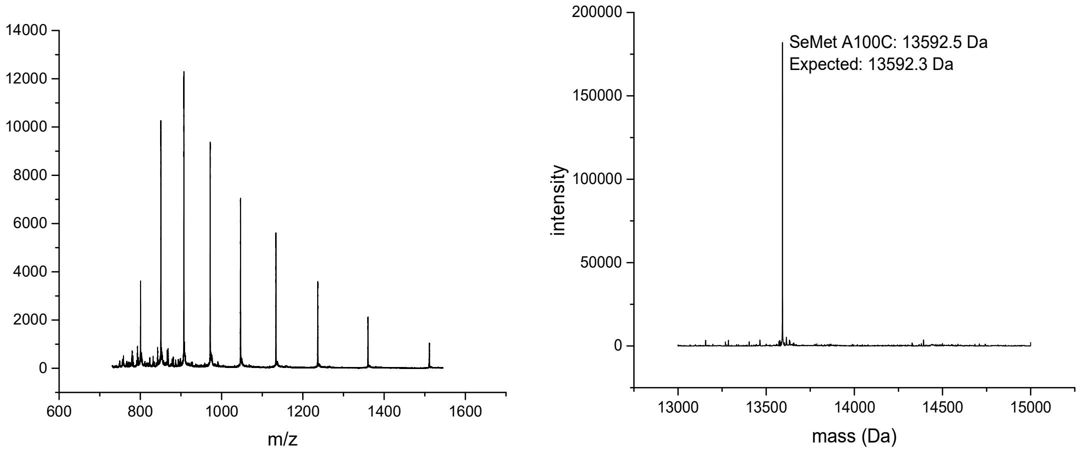

Figure S2: Raw mass spectrum of SeMet A100C (left) and the deconvoluted mass-spectrum of SeMet A100C (right) obtained by LC-MS (ES+).Pictures created using Origin.

Alanine mutants:

The alanine mutants of SCP-2L A100C, M1A, M80A, M105A and M112A were introduced using site directed mutagenesis using the Huanting's method.[3]

Primer	Mutation acquired	Sequence
**Primer 1 fw**	M1A ATG/GCA	*AGGGCGCc**GCA**gaGG*GAGGGAAGCTTCAGAGtACC
**Primer 1 rv**		*CCtc**TGC**gGCGCCCT*GAAAAtACAGGTTTTCGGTcgt
**Primer 2 fw**	M80A ATG/GCA	*AAGATTTC**GCA**GAGGTgg*tcCTGGGCAAGCTTGACCC
**Primer 2 rv**		*ccACCTC**TGC**GAAATCTT*CATCTGAAAGTATGATTGTGTATC
**Primer 3 fw**	M105A ATG/GCC	*CATC**GCC**C*TGAGCCAGAAACTTCAG
**Primer 3 rv**		*G**GGC**GATG*TTCCCTCTGCACTTCAG
**Primer 4 fw**	M112A ATG/GCA	*TCAG**GCA**ATTCTTAAAGAC*TATGCCAAGCTCTGAGGATCC
**Primer 4 rv**		*GTCTTTAAGAAT**TGC**CTGA*AGTTTCTGGCTCAGCATGATG

After successful mutagenesis confirmed by sequencing, the proteins were obtained using the same procedure described for SCP-2L A100C and in the following yields and all gave the expected mass:

SCP-2L A100C M1A: 38 mgL^{-1} media
SCP-2L A100C M80A: 11 mgL^{-1} media
SCP-2L A100C M105A: 52 mgL^{-1} media
SCP-2L A100C M112A: 11 mgL^{-1} media

2. Protein Modification

Chemicals were purchased in highest commercially available purity unless stated otherwise. 3-Maleimidopropionic acid hydrazide hydrochloride, **1**, and 2-(diphenylphosphino)benzaldehyde, **P1**, were obtained from Apollo Scientific Ltd and Aldrich respectively. 3-(Diphenylphosphino)benzaldehyde, **P2**[4], and 4-(diphenylphosphino)benzaldehyde, **P3**[5], were synthesised following literature procedures. The phosphine selenide **P3(Se)** was prepared by reaction of the phosphine aldehyde (1 eq.) with Se metal (1.25 eq.) in toluene at rt. The phosphine selenide was obtained by filtration of the excess Se and removal of the solvent. ^{31}P ($CDCl_3$, 162 MHz) δ 35.2 (J_{PSe} = 741 Hz). **P3-AuCl** was prepared via reaction of the

P3 (1 eq.) and $Au(SMe_2)_2Cl$ (1 eq) in DCM at rt overnight. Removal of the DCM gave the **P3**AuCl complex as a yellow solid. ^{31}P (DCM, 162 MHz) δ 30.9 ppm.

All reactions involving phosphines were performed under an argon atmosphere using degassed solvents and standard Schlenk techniques. Buffer solutions were degassed by bubbling argon through the solution. Protein solutions were degassed by washing and concentrating three times with degassed buffer using a filter unit set up as shown in Figure S3. Protein concentrations were determined by Bradford's reagent.[6]

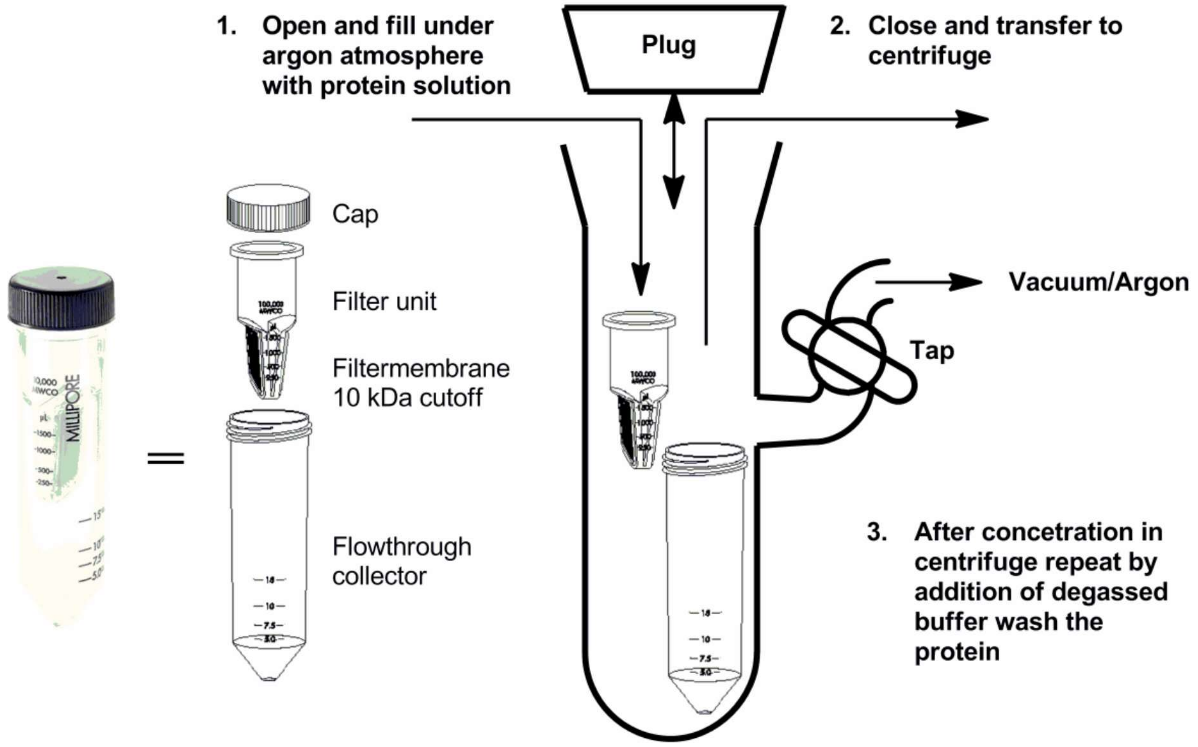

Figure S3: Application of Amicon® Ultra-15 Centrifugal Filter Unit under inert atmosphere using a large custom made Schlenk flask.

Modification of the protein scaffold was carried out according to literature procedures.[7] For the rhodium complex formation the Rh:P ratio aimed for depended on application. For characterisation a 1 : 1 ratio was used. For catalysis an excess of phosphine, approximately 2 equiv. per Rh was used to ensure that no 'free Rh' is present. Free Rh-catalysed hydroformylation is characterised by high TON's and low branched to linear selectivities. Slight inconsistencies in protein concentration, through loss in handling or oxidation of the phosphine have the potential to lead to excess Rh over phosphine if a 1:1 ratio is used. $Rh(acac)(CO)_2$ was used as the rhodium source, and added as a freshly made solution in DMF.

SCP-2L A100C-**1**-**P3**-**AuCl**:

To a solution of SCP-2L A100C-**1** (13 mg, 71 μM in degassed Mes 50 mM, NaCl 20 mM, pH 6 buffer, 9.57×10^{-7} mol) was added **P3-AuCl** (2.5 mg in 151 μL degassed DMSO, 4.78×10^{-6} mol, 5 eq.). The reaction mixture was shaken under Ar at 80 rpm at rt. After 5 h the MS showed incomplete conversion, so a further 5 eq. of **P3-AuCl** were added. After a further 48 h, the excess complex was removed by buffer exchange (degassed Mes 50 mM, NaCl 20 mM, pH 6 buffer) using centrifugal concentrators (MWCO 10 kDa) to give 10.2 mg of protein (75% yield). LC-MS showed the desired ArM, alongside some free phosphine and complex adducts. LC-ME (ESI^+) m/z 14057 Da (SCP-2L A100C-**1**-**P3**-**Au** calc. 14057) Da.

SCP-2L A100C-**1**-**P3(Se)**:

To a solution of SCP-2L A100C-**1** (14 mg, 105 µM in Mes 50 mM, NaCl 20 mM, pH 6 buffer, 1.05 x 10^{-6} mol) was added **P3(Se)** (3.9 mg in 100 µL MeCN, 1.05 x 10^{-5} mol, 10 eq.) and the reaction mixture shaken overnight at rt. The excess **P3(Se)** was removed by buffer exchange using centrifugal concentrators (MWCO 10 kDa). MS showed full conversion and the ArM was obtained in quantitative yield. LC-MS (ESI$^+$) m/z 13940 Da (SCP-2L A100C-**1**-**P3(Se)** calc. 13939 Da).

3. Characterisation of the modified proteins

3.i Mass spectrometry on intact proteins and modified proteins:

LC-MS(ES$^+$) used for analysis of protein and conjugation-reactions was performed on a Waters Alliance HT 2795 equipped with a Micromass LCT-TOF mass spectrometer, using positive electrospray ionisation and applying a Waters MASSPREP® On-line Desalting 2.1x10 mm cartridge using a gradient of 1% formic acid in H_2O to 1% formic acid in acetonitrile. ESI-MS results were analysed by MassLynx V. 4.0 and its MaxEnt algorithm.

The samples for phosphine modified proteins were prepared immediately before injection to reduce the oxidation of the sample.

A100C modification:
A representative example of the mass spectra obtained on modification of A100C is shown below for **P3**:

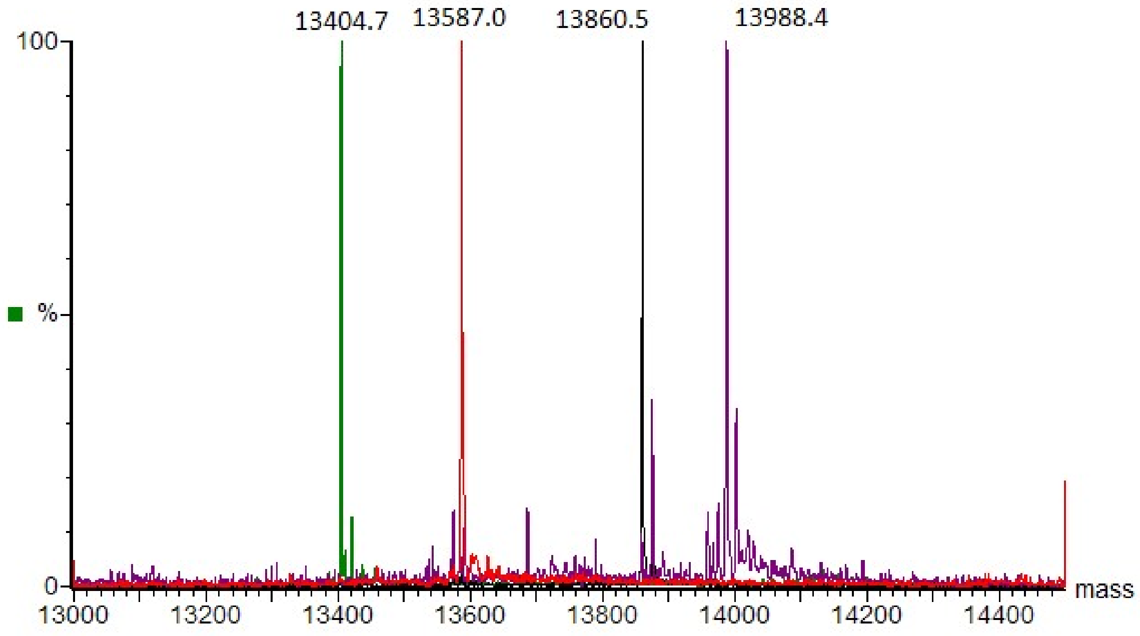

Figure S4: Deconvoluted mass-spectrum of modified A100C proteins: Green: A100C 13404.7 Da (Calc.13404.6 Da).Red: A100C-**1** 13587.0 Da (Calc. 13587.9 Da). Black: A100C-**1**-**P3** 13860.5 Da (Calc. 13860.2 Da).Purple: A100C-**1**-**P3**-Rh(CO) 13988.4 Da (Calc. 13991.1 Da) obtained by LC-MS (ESI$^+$).

SeMet-A100C modification with **P3**:

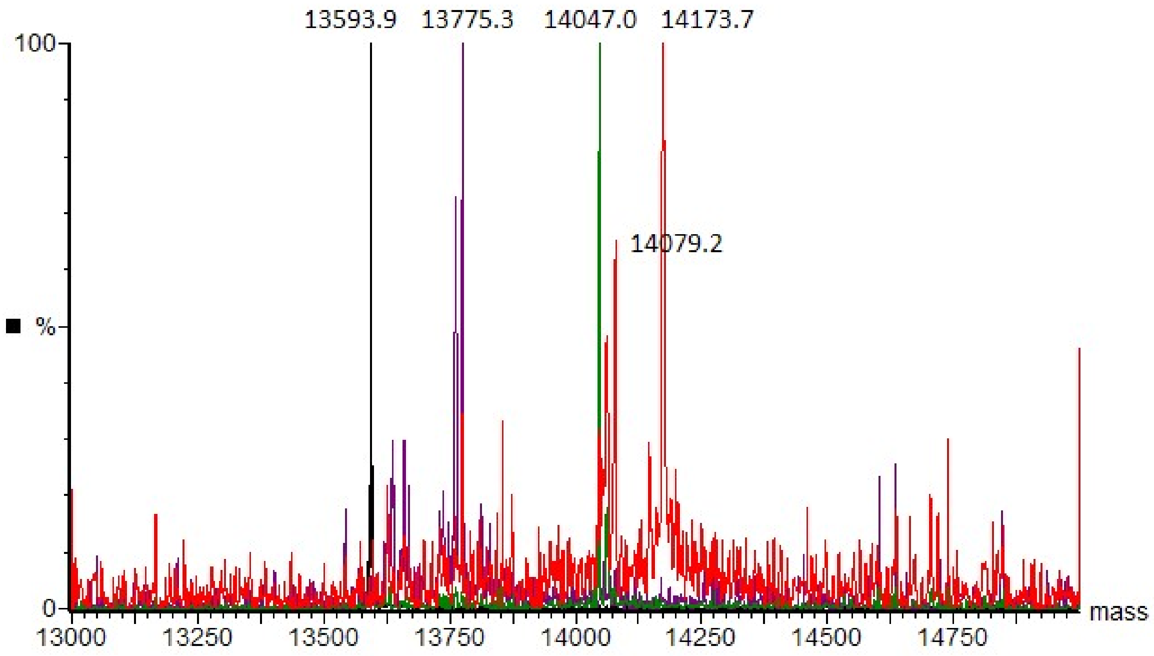

Figure S5: Deconvoluted mass-spectrum of modified A100C proteins. Black: SeMet A100C 13593.9 Da (Calc. 13592.3 Da). Purple: SeMet A100C-**1** 13775.3 Da (Calc. 13775.4 Da). Green: SeMet A100C-**1**-**P3** 14047.0 Da (Calc. 14047.7 Da). Red: SeMet A100C-**1**-**P3**-Rh(CO) 14173.7 Da (Calc. 14178.6), 14079.2 (SeMet A100C-**1**-**P3** + 2O – attributed to methionine and phosphine oxidation) obtained by LC-MS (ESI^+).

An example of the alanine mutants modification showing the results for SCP-2L A100C M1A modification:

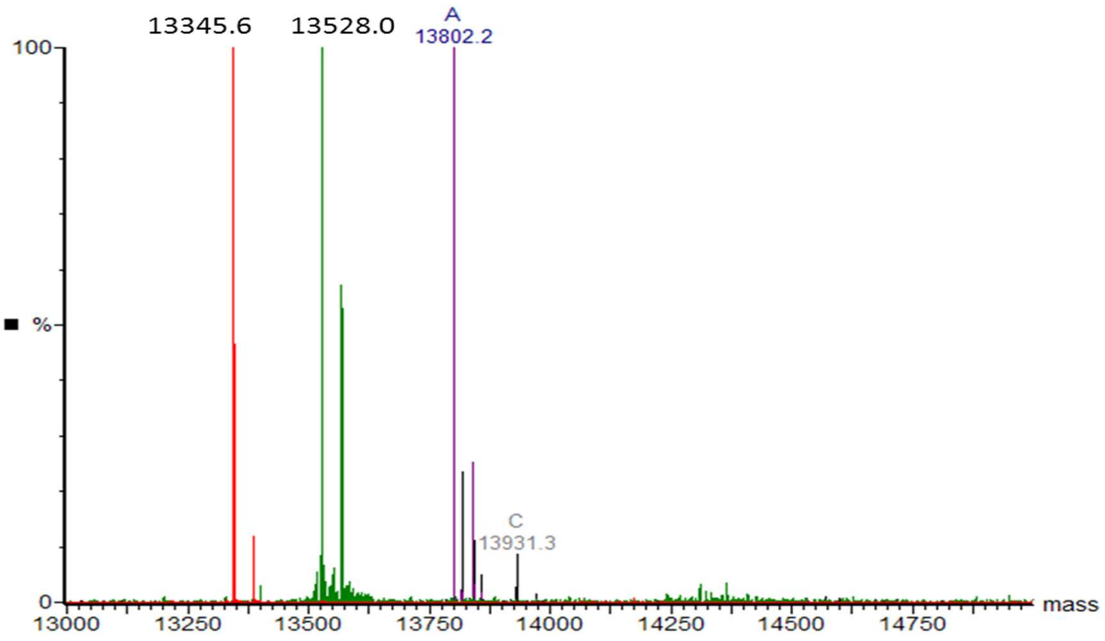

Figure S6: Deconvoluted mass-spectrum of modified A100C proteins. Red: A100C M1A 13345.6Da (Calc.13344.6 Da). Green: A100C M1A-**1** 13528.0 Da (Calc. 13527.7 Da). Purple: A100C M1A-**1**-**P3** 13802.2 Da (Calc. 13800.0Da). Black: A100C M1A-**1**-**P3**-Rh(CO) 13931.3 Da (Calc. 13930.9 Da), (A100C M1A-**1**-**P3** + O 13818.2 Da – attributed to methionine and/or phosphine oxidation) obtained by LC-MS (ESI^+).

3.ii. CD:

Circular dichroism spectra were obtained using a Biologic MOS-500 spectrometer. A Xe lamp was used, and the near UV spectra taken from 190 – 260 nm using a 10 mm pathlength cell. The other parameters were as follows: 0.25 nm step, 0.5 s acq period, 3 repeats, ±MD 30 and slit size 2 nm.
The CD spectra were taken on samples at a concentration of between 0.5-1.5 mg/mL and were corrected for concentration according to the following equation to give the molar ellipticity [θ] values:

$$[\theta]\ (\mathrm{degcm^{2}dmol^{-1}}) = (\mathrm{mD\ sample} - \mathrm{mD\ blank}) / 10\ {*}\mathrm{conc.\ (M)}{*}\mathrm{pathlenght\ (cm)})$$

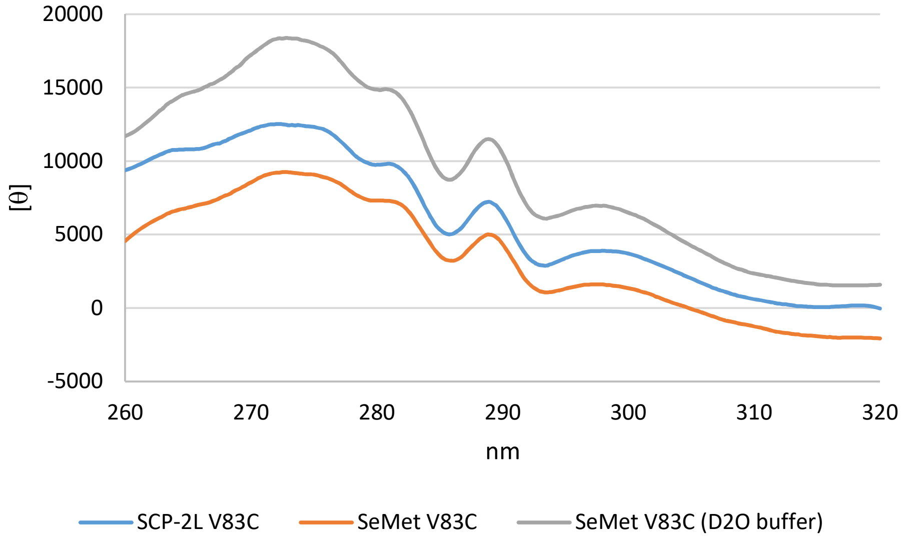

Figure S7: Near CD spectra of SCP-2L V83C and SeMet V83C in 20 mM MES, 50 mM NaCl, pH 6.

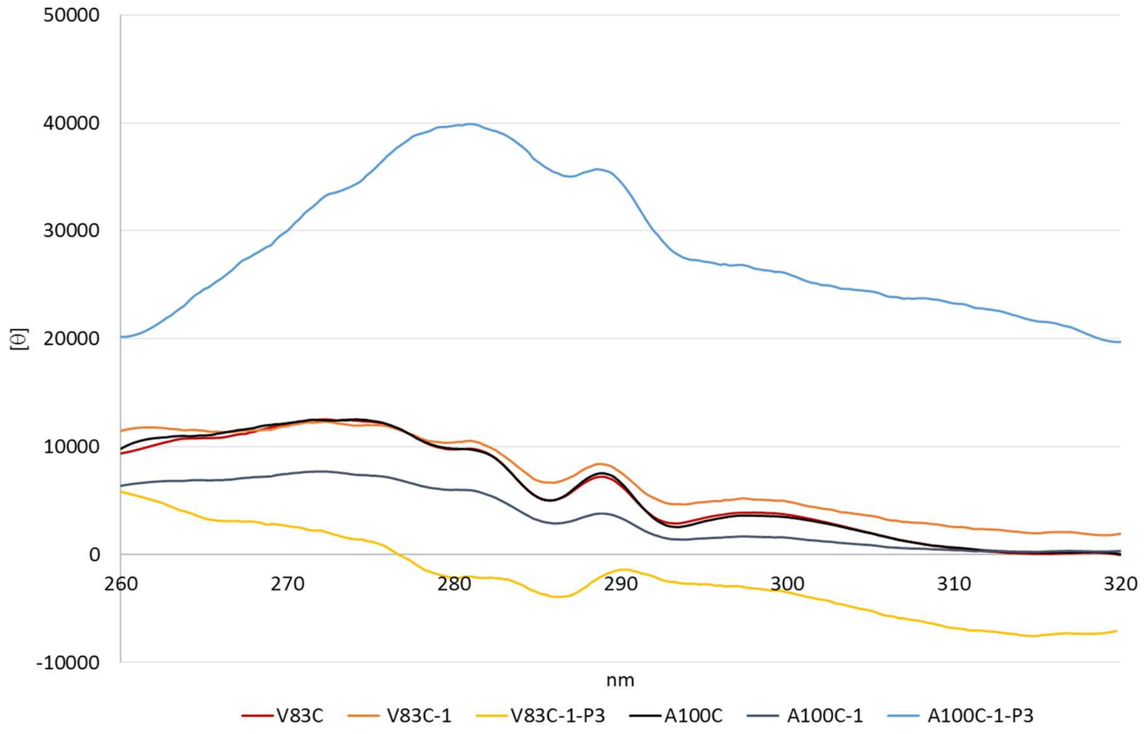

Figure S8: Changes in Near UV CD spectra on modification of SCP-2L V83C and A100C (20 mM MES, 50 mM NaCl, pH 6).

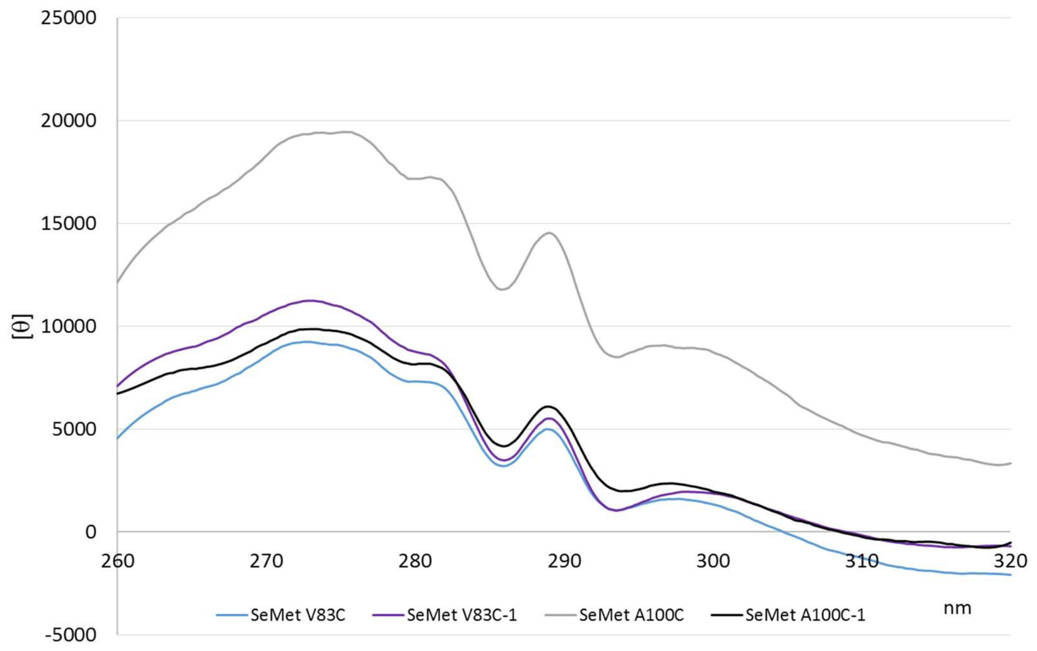

Figure S9: Comparison of Near UV CD spectra of SeMet proteins and hydrazide modified SeMet proteins (20 mM MES, 50 mM NaCl, pH 6).

3.iii. XAFS:

The protein samples for EXAFS were prepared as described in section 2. The samples were then concentrated to about 5 mM, before transferring 120 μL of solution into a gelatine capsule (size 4 Elektron Technology (Agar Scientific Limited)) and flash frozen immediately in liquid nitrogen. The samples were stored in liquid nitrogen until the experiment, where they were transferred to a cryostat (78K) mounted on the beamline.

XAFS experiments were performed on B18 at Diamond Light Source, Harwell using a QEXAFS set-up and a fast-scanning Si (311) double crystal monochromator. The protein samples were measured in fluorescence mode using a 36 element solid state Ge detector (K_{max} = 15), where 70 acquisitions (2.5 min per scan) were acquired to improve the signal to noise. The reference compounds were diluted with cellulose and pressed as pellets to achieve samples for transmission measurements with an appropriate edge-step. Transmission measurements were performed with ion chamber detectors, where, on average, 3 x 1 minute scans (K_{max} = 17) where recorded to achieve satisfactory data quality. XAS data processing was performed using the Demeter IFEFFIT package.[8, 9]

Table S1: EXAFS Fitting parameters

	Abs. Sc.	N	R /Å	2σ² / Å²	E_f / eV	R_{factor}
SCP-2L A100C-**1**-**P3**-Rh	Rh-O (acac)	2 (fixed)	2.10 (1)	0.001	7 (2)	0.02
	Rh-P	2 (fixed)	2.40 (1)	0.026(6)		
	Rh-C	2 (fixed)	2.80 (2)			
SeMet SCP2-L A100C-**1**-**P3**-Rh	Rh-O (acac)	2 (fixed)	2.08 (1)	0.002 (1)	0 (fixed)	0.03
	Rh-P	1 (fixed)	2.34 (fixed)	0.001 (0)		
	Rh-Se	1 (fixed)	2.49	0.001 (0)		
	Rh-C	4 (fixed)	3.11	0.002 (2)		

Fitting parameters: S_0^2 = 0.81; Fit range 3.5<k<13, 1<R<3; # of independent points = 11

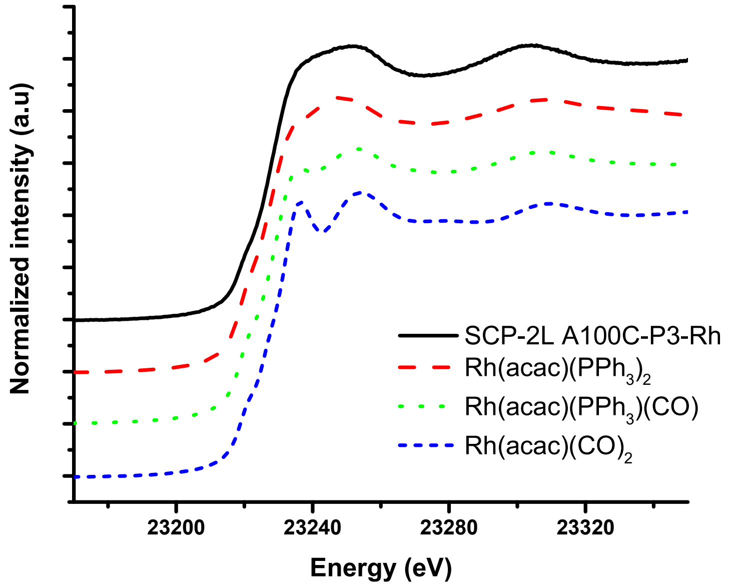

Figure S10: Normalized Rh K edge XANES spectra for A100C-**1-P3**-Rh, Rh(acac)(PPh_3)$_2$, Rh(acac)(PPh_3)CO, and Rh(acac)(CO)$_2$.

ICP analysis was used to determine the exact phosphorous and rhodium concentrations in the EXAFS samples. A rhodium concentration of 5 mM was aimed for and a 1 : 1 ratio of P : Rh expected. Trace metal analysis was performed by the microanalysis service at the University of Edinburgh on a Perkin Elmer Optima 5300 DV ICP-OES.

Sample preparation: To a 30 µL aliquot in a 15 mL falcon tube was added nitric acid (200 µL, 69% Aristar for trace analysis) and heated. The protein fully dissolved giving a yellow solution that was then diluted to 3 mL. The samples were not filtered before analysis.

Table S2: Phosphorous and rhodium concentrations of the artificial hydroformylase

Construct	P conc. (mM)	Rh conc. (mM)	Ratio P : Rh
**SCP-2L A100C-1-P3-Rh**	1.91 (0.24)	2.32 (0.16)	0.83 : 1
**SeMet SCP-2L A100C-1-P3-Rh**	3.47 (0.71)	3.10 (0.15)	1.12 : 1

a) Obtained by ICP-OES analysis. (Standard deviation in brackets from two separate ICP runs)

3.iv. NMR:

^{1}H-^{77}Se HMBC (optimised for J = 8 Hz) was used to determine the Se shifts of the protein using a Bruker AVIII-HD 500 instrument with a SmartProbe BBFO+. $SeMe_2$ was used as the reference for ^{77}Se. The protein was exchanged into buffer made with D_2O and concentrated to ~0.5 mM.

SeMet SCP 2LA100C (500 MHz, 0.5 mM in 20 mM MES, 50 mM NaCl, pH 6, D_2O) ^{77}Se δ 64 ppm.
SeMet SCP2L A100C-**1**-**P3** (500 MHz, 0.51 mM in 20 mM MES, 50 mM NaCl, pH 6, D_2O) ^{77}Se δ 64 ppm.
SeMet SCP2-L A100C-**1**-**P3**-Rh (500 MHz, 0.57 mM in 20 mM MES, 50 mM NaCl, pH 6, D_2O) ^{77}Se δ 60, 64 ppm.

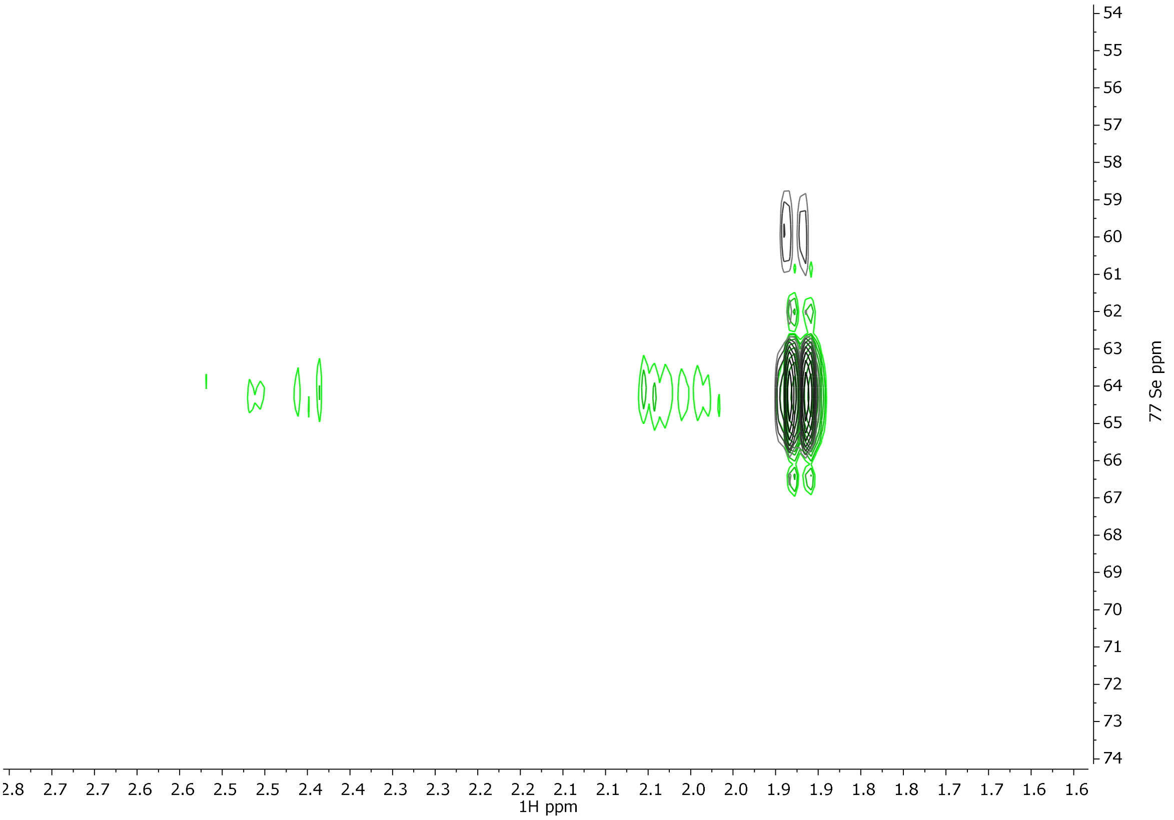

Figure S11: ^{1}H-^{77}Se HMBC spectra. Green spectrum: SeMet SCP-2L A100C-**1**-**P3** (0.51 mM). Grey spectrum: SeMet SCP-2L A100C-**1**-**P3**-Rh (0.57 mM).

3.v. *Infra-red studies:*

Infra-red studies were undertaken on an IR Affinity–IS FT IR spectrometer (Shimadzu) using Lab solutions software. Samples with concentrations of about 2 mM were measured using a 0.1 mm pathlength solution cell (CaF_2), between 1700-2900 cm^{-1}, 0.5 cm^{-1} resolution, 250 scans. No evidence of CO stretches were observed under these conditions, whilst for 2 mM and 0.2 mM solutions of Rh(acac)(CO)(PPh_2) in DCM the CO stretch was observed (1977 cm^{-1}).

3.vi. *Protein binding studies:*

A fluorescent binding assay was performed according to literature procedures[10] using pyrenedodecanoic acid (Pyr-C12) as described previously.[11] Similar binding constants were obtained for the WT protein, V83C mutant and the modified V83C. Note: the binding was not carried out under an inert atmosphere thus for SCP-2L V83C-**1**-**P3** the actual species is likely to be a mix of phosphine and phosphine oxide.

Table S3: Binding constants of Pyr-C12 to different protein scaffolds.

Protein	K_d, µM
**SCP-2L**	0.18 (±0.05)
**SCP-2L V83C**	0.18 (±0.07) [6]
**SCP-2L V83C-1-P3**	0.24 (±0.06)

3.vii. *Alternate Rh precursors:*

The complexation of SCP-2L A100C-**1**-**P3** with different rhodium precursors was studied.
The Rh precursor (3 eq.) was added to the modified protein (0.07 µmol/mL). The mixture was occasionally shaken over the course of 1 day. The buffer was exchanged (3 times, from >10 mL to <1 mL). Analysis by LC-MS and ICP-MS was performed. The metal loading is quoted at concentration of Rh compared to protein concentration determined by Bradford reagent.

Table S4: Data on complexation with alternate Rh precursors

Rh precursor	MS	Metal loading, %Rh
**[Rh(MeCN)$_2$COD]BF$_4$**	+99 Da (+Rh)	226%
**RhH(PPh$_3$)$_3$CO**	No Rh adducts observed	77%
**Rh(acac)(CO)$_2$[a]**	+128 Da (+RhCO)	120%

[a]Results obtained from the XAFS samples formed from the addition of 1 eq. of Rh and quoted vs phosphorous concentration – see Section 3iii.

For RhH(PPh_3)$_3$CO the resulting protein mixture showed a metal loading of 77%, coupled with the lack of an identifiable Rh mass adduct this suggests unselective binding of the Rh to the protein scaffold. Results with carbonic anhydrase and Rh precursors showed that cationic complexes were less selective when binding and would bind to the protein surface,[12] a result that matches our observations which show a Rh loading of 226% when using the [Rh(MeCN)$_2$COD]BF_4.

4. Hydroformylation

4.i. General hydroformylation procedure:

Hydroformylation reactions were carried out in stainless steel autoclaves (up to two simultaneously) containing up to eight glass reaction vials (volume of approximately 5 mL) each. The reaction vials were equipped with stirring bars as well as a septum cap pierced with a needle in order to allow contact with the reaction gases. The prepared vials were placed in an autoclave which was flushed three times with >20 bar argon before adding the chemicals. The vials were charged under an argon blanket. The aqueous Rh-enzyme solution was added to each vial (0.5 mL, 20 mM MES, 50 mM NaCl, pH = 6). A protein-P : Rh ratio of 2 : 1 was aimed for and an aliquot of this solution was saved for ICP-MS analysis to obtain the actual Rh concentration. An organic solution consisting of 1-alkene, 9% (v/v) heptane and 1% (v/v) diphenyl ether as internal standards, freshly filtered over activated alumina (dried under vacuum at 150 °C for at least 5 hours) to remove peroxides, was added (0.5 mL). The autoclave was then flushed 3 times with about 20 bar syngas (H_2 : CO 1 : 1) and subsequently charged to the desired pressure (80 bar). The autoclave was then placed into an oil bath that was preheated to the desired temperature (35 °C) and pre-set to the desired stirring speed (625 rpm). After the reaction time (48 h) the reaction was stopped by putting the autoclave on ice for at least 15 mins and slowly releasing the pressure. The organic phase was analysed by GC, and ICP-MS for metal leaching. All experiments were run in quadruple, unless otherwise stated.

Table S5: Results for the hydroformylation of linear alkenes with Rh-ArM's.

Entry	Catalyst	1-octene		1-decene		1-dodecene		1-octadecene	
		TON	% linear aldehyde	TON	% linear aldehyde	TON	% linear aldehyde	TON	% linear aldehyde
1	SCP-2L V83C-**1**-**P1**-Rh[a]	0.9 (0.26)	*68.6 (1.14)*	nd	*nd*	nd	*nd*	nd	*nd*
2	SCP-2L V83C-**1**-**P2**-Rh[b]	33.3 (2.07)	*74.1 (0.21)*	27.1 (6.75)	*71.8 (0.17)*	6.0 (2.67)	*73.5 (0.80)*	3.8 (0.75)	*75.5 (1.28)*
3	SCP-2L V83C-**1**-**P3**-Rh[c]	74.5 (8.68)	*78.3 (0.29)*	39.0 (7.00)	*76.3 (0.48)*	13.4 (5.08)	*75.3 (1.61)*	1.5 (0.71)	*70.7 (2.94)*
4	SCP-2L A100C-**1**-**P1**-Rh[d]	1.2 (0.55)	*69.9 (3.58)*	nd	*nd*	nd	*nd*	nd	*nd*
5	SCP-2L A100C-**1**-**P2**-Rh[e]	105.8 (2.15)	*69.9 (0.95)*	75.2 (10.45)	*66.4 (0.95)*	45.8 (9.23)	*64.1 (0.27)*	21.0 (4.41)	*63.6 (1.31)*
6	SCP-2L A100C-**1**-**P3**-Rh[f]	408.7 (57.79)	*78.8 (4.86)*	134.6 (15.75)	*74.0 (0.90)*	66.5 (3.22)	*72.9 (0.39)*	20.3 (7.42)	*71.9 (1.59)*
7	SCP-2L A100C + Rh and washed[g, h]	123.5 (38.19)	*57.8 (0.07)*						
8	WT SCP-2L + Rh washed[g, i]	585.7 (122.33)	*56.8 (0.27)*						
9	SCP-2L A100C-**1**-**P3Se**[j]	4.3 (0.88)	*55.2 (1.22)*						
10	SCP-2L A100C-**1**-**P3**-**Au**[k]	5.4 (1.36)	*54.0 (1.77)*						

Standard conditions: 80 bar syn gas (1:1), 35 °C, stirring 625 rpm, 0.5 mL of catalyst solution and 0.5 mL of alkene containing 9% (v/v) n-heptane and 1% (v/v) diphenyl ether, 48 h. Rh concentration was obtained by ICP-MS and used to calculate TON. Conversions and linear selectivities were obtained by GC using a minimum of 3 runs. Standard deviation in brackets.a P:Rh 5.8, 30.9 nmol Rh. b P:Rh 1.8, 99.1 nmol Rh. c P:Rh 3.0, 46.6 nmol Rh. d P:Rh 3.5, 26.9 nmol Rh. e P:Rh 2.2, 37.7 nmol Rh. f P:Rh 1.5, 23 nmol Rh. Green: TON >100, l:b >3 (75%); Yellow: TON >10<100, l:b >2.3 (70-75%); Orange: TON <10, l:b <2.3 (70%). nd = not detected. g. Treated with Rh and washed in the same manner as the metalloproteins. h Rh 10.0 nmol, i 9.08 nmol, j Turnover based on Se concentration: 48 nmol Se, k Turnover based on Au concentration: 50.5 nmol Au.

Control reactions showed that the Rh-metalloprotein is responsible for the hydroformylation (entries 7-10, Table S5). To show that the rhodium is not binding to another site in the protein scaffold, SCP-2L A100C and WT SCP-2L were treated with a solution of Rh(acac)$(CO)_2$ and then washed with buffer using the centrifugal concentrators in the same manner as the metalloproteins. Only low selectivity was observed (entries 7 and 8, Table S5) suggesting that residual weakly bound Rh remained after washing, which acted as free rhodium. In addition, both the phosphine selenide and gold complex (preparation in section 2) showed negligible hydroformylation activity (entries 9 and 10, Table S5) and low selectivity. The presence of ppb levels of Rh contamination cannot be ruled out and could be responsible for the observed turnover.[13]

Table S6: Relative activity (RA) of the hydroformylation of 1-octene over time using SCP-2L V83C-**1**-**P3**-**Rh**[a]

Relative activity (RA) with 1-octene across time					
3.0 h		***6.5 h***		***16.0 h***	
**RA**	**l:b**	**RA**	**l:b**	**RA**	**l:b**
100.0 (3.6)	*3.64 (0.18)*	117.6 (10.3)	*3.55 (0.11)*	94.0 (11.2)	*3.65 (0.03)*

a. Standard conditions: 80 bar syn gas (1:1), 35 °C, stirring 625 rpm, 0.5 mL of catalyst solution and 0.5 mL of alkene containing 9% (v/v) n-heptane and 1% (v/v) diphenyl ether. ICP not obtained therefore relative activity used.

The activity of the protein remains constant for the first 16 h of the reaction (see Table S6). In addition, comparing the TON after 16 h and 49 h for one batch of protein showed that the catalyst was still active, decreasing from an overall TOF 7 h^{-1} to 5 h^{-1} over 2 days.

4.ii. Hydroformylation using phosphine ligands:

The desired amount of Rh(acac)$(CO)_2$ (1 eq.) and TPPTS * 3 Na *4 H_20 (2-300 eq.) were dissolved in degassed methanol (5-10 mL) at room temperature for 2-4 hr. The solvent was removed and the solid redissolved in degassed buffer (10 mL; 0.6 mM Rh; 20 mM MES, 50 mM NaCl, pH = 6) to give a stock solution of catalyst for the reaction. Hydroformylation was carried out as described by the general procedure above.

Table S7: Hydroformylation using a biphasic Rh/TPPTS system.

Entry	Rh (nmol)	Rh:TPPTS ratio	1-octene		1-decene		1-dodecene		1-octadecene	
			TON	% linear aldehyde	TON	% linear aldehyde	TON	% linear aldehyde	TON	% linear aldehyde
**1**	300	1:1	427 (77)	*1.26[d] (0.01)*	667 (170)	*1.27[d] (0.02)*			223 (65)	*1.27[d] (0.01)*
**2**	260.6	1:30	1.0 (0.86)	*72.2 (7.31)*	0.04[c] (0.01)	*72.6 (2.42)*	nd	*nd*	nd	*nd*
**3**	30	1:2	2245 (674)	*58.9 (0.41)*						
**4**		1:5	1457 (141)	*56.9 (0.61)*						
**5**		1:10	1579 (228)	*56.4 (0.07)*						
**6**		1:20	700 (190)	*56.5 (0.08)*						
**7**		1:300	5.4 (3.25)	*65.9 (8.55)*						

**8**		1:3000	UDL[c]	*UDL[c]*						
**9**	30	1:300[a]	5.1 (2.48)	*67.0 (0.64)*						
**10**	30	1:300[b]	9.4 (1.01)	*60.7 (1.48)*						

Standard conditions: 80 bar syn gas (1:1), 35 °C, stirring 625 rpm, 0.5 mL of catalyst solution and 0.5 mL of alkene containing 9% (v/v) n-heptane and 1% (v/v) diphenyl ether, 48 h. Rh concentration was obtained by ICP-MS and used to calculate TON. Conversions and linear selectivities were obtained by GC using a minimum of 3 runs. Standard deviation in brackets. Green: TON >100, l:b >3 (75%); Yellow: TON >10<100, l:b >2.3 (70%); Orange: TON <10, l:b <2.3 (70%). nd = not detected. [a] 2 eq. WT SCP-2L. [b] 2 eq. SCP-2L A100C. c. Product was observed but the amounts were at the detection limit and therefore not quantifiable. [d] results recorded as l:b ratios, equivalent in % is 55%.

When a low number of equivalents of phosphine ligand are used results indicating that 'free Rh' is present in the organic layer are observed (high TON, low selectivity). Increasing the equivalents to 300 decreases the TON and increases the selectivity (Entry 1 vs 2, Table S7), implying that the equilibrium shifts towards the bis-ligated phosphine rhodium complex, which gives good selectivity, but lower TON.[14] When a similar concentration of Rh is used as in the ArM reaction (~30 nmol, entries 3-8, Table S7) even 300 equivalents of TPPTS is not enough to switch the selectivity to what was observed with the ArMs.

To investigate if the protein itself acted just as a phase transfer reagent the unmodified protein scaffold was added to the Rh/TPPTS reaction (entry 9 and 10, Table S7). Under these conditions the TON did not change significantly. If a Rh(acac)(CO)(PPh_3) complex was used in the hydroformylation reaction the same results (TON ~100 and selectivity ~70%) were obtained in a single phase organic reaction as in the biphasic reaction containing the protein scaffold (Table S8). This indicates that the activity in the biphasic system is not due to neutral complex binding within the protein scaffold, but just leaching into the organic layer.

Table S8: Hydroformylation with Rh(acac)(CO)(PPh_3) under various conditions.

Entry	Rh (nmol)	Rh:PPh_3 ratio	1-Octene	
			TON	% linear aldehydes
**1[a]**	52	1:1	145.7 (6.02)	72.1 (0.81)
**2[b]**	15	1:1	223.5 (18.16)	69.5 (1.50)

Standard conditions: 80 bar syn gas (1:1), 35 °C, stirring 625 rpm, 0.5 mL of alkene containing 9% (v/v) n-heptane and 1% (v/v) diphenyl ether. Rh concentration was obtained by ICP-MS and used to calculate TON. Conversions and linear selectivities were obtained by GC using a minimum of 2 runs. Standard deviation in brackets. a) Rh(acac)(CO)(PPh_3) added from a DMF stock solution (5µL, 10.4 mM). b) Rh(acac)(CO)(PPh_3) added from a DMF stock solution to a solution of WT SCP-2L (2 eq.) in buffer (20 mM MES, 50 mM NaCl, pH 6) and 0.5 mL of this solution added to the reaction.

In order to show that the activity was not due to breakdown of the hydrazine and leaching of the phosphine aldehyde and Rh into the organic layer test reactions were performed using the phosphine aldehydes (**P1-3**) and Rh(acac)($CO)_2$ in 1-octene (Table S9). It was observed that to see the same selectivity as observed with the ArM reactions over 1 eq of aldehyde would need to be in the organic layer i.e. all the ArM would have degraded. Looking at the protein after the reaction (Figure S11) no large amounts of degradation products are observed instead mostly oxidised ArM is observed: SCP-2L A100C-**1-P3(O).**

Table S9: Hydroformylation of 1-octene with the different phosphine aldehydes at varying Rh:P ratios (constant Rh) in organic phase (1-octene).[a]

Entry	Phosphine	Rh : P[b]	TON[c]	% linear selectivity[c]
1	P1	0.15 : 0	312 ± 36	56 ± 0
2		0.15 : 0.08	728 ± 72	56 ± 0
3		0.15 : 0.21	386 ± 47	56 ± 0
4		0.15 : 1.6	1 ± 0	68 ± 1
5	P2	0.15 : 0	530 ± 53	55 ± 1
6		0.15 : 0.05	691 ± 58	56 ± 0
7		0.15 : 0.30	311 ± 27	74 ± 1
8		0.15 : 1.7	521 ± 33	76 ± 0
9	P3	0.14 : 0.02	467 ± 73	57 ± 0
10		0.14 : 0.08	486 ± 91	58 ± 1
11		0.14 : 0.16	238 ± 17	74 ± 2
12		0. 14 : 1.5	487 ± 43	78 ± 0

a. Conditions: 80 bar syn gas (1:1), 35 °C, stirring 625 rpm, Rh(acac)CO_2 and phosphine aldehyde added in the appropriate amounts from stock solutions in 1-octene containing 9% (v/v) n-heptane and 1% (v/v) diphenyl ether, made up to a total volume of 0.4 mL 1-octene solution, 41.25 h. b. Actual molar amounts used in µmol. c. Conversions and linear selectivities were obtained by GC, each entry represents the average of 4 runs.

4.iii. Hydroformylation of 1-octene with alanine mutants

Hydroformylation experiments were carried out according to the general procedure (see Section 4.i) over 16 h. It is unclear from these results alone if the large error for M105A is due to M105 playing a role in stabilising the Rh or if this is just due to increased instability of the protein scaffold on replacement of Met with Ala.

Table S10: Hydroformylation of 1-octene using the alanine mutants of A100C

Entry	Catalyst	TON	%linear selectivity
1	SCP-2L A100C-**1**-**P3**	60.9 (5.40)	77.4(3.27)
2	SCP-2L A100C M1A-**1**-**P3**	86.0 (9.16)	80.8 (1.04)
3	SCP-2L A100C M80A-**1**-**P3**	67.4 (6.77)	76.1 (0.49)
4	SCP-2L A100C M105A-**1**-**P3**	112.1 (33.1)	74.4 (2.37)
5	SCP-2L A100C M112A-**1**-**P3**	66.7 (5.04)	75.5 (0.51)

Standard conditions: 80 bar syn gas (1:1), 35 °C, stirring 625 rpm, 0.5 mL of catalyst solution and 0.5 mL of alkene containing 9% (v/v) n-heptane and 1% (v/v) diphenyl ether, 16 h.. Rh concentration was obtained by ICP-MS and used to calculate TON. Conversions and linear selectivities were obtained by GC using a minimum of 3 runs. Standard deviation in brackets

4.iv. Gas chromatography:

A CE instruments GC8000 Top equipped with a AS800 autosampler was used for GC analysis. The GC was installed with a Restek RTX-1 column 30 m x 0.25 mm x 0.1 µm. Conditions: injector temperature 250 °C; 100 kPa constant pressure; 100:1 split ratio; 1 or 2 µL injection depending on the signal strength;

oven method, 45 °C isotherm for 5 min, 20 °C/min to 130 °C, 130 isotherm for 2 min, 20 °C/min to 250 °C, 250 °C isotherm for 10 min and 20 °C/min to 300 °C. Retention times: 1-hexene 2.1 min, heptane 2.6 min, 1-octene 3.7 min, 2-methylhexanal 4.8 min, *n*-heptanal 5.7 min, 1-decene 7.6 min, 2-methyloctanal 8.3 min, *n*-nonanal 8.7 min, 1-dodecene 9.7 min, 2-methyldecanal 10.3 min, *n*-undecanal 10.7 min, diphenyl ether 11.8 min, 2-methyldodecanal 12.6 min, *n*-tridecanal 13.0 min, 1-octadecene 15.2 min, 2-methyloctadecanal 16.6 min, *n*-nonadecanal 16.6 min.

Alternatively, an Agilent 7820A GC system with autosampler was used for GC analysis. This GC was installed with an Agilent HP-5 column 30m x 0.32 mm x 0.25 µm. Conditions: injector temperature 250 °C; FID detector temperature 300 °C; 6.5 mL/min constant flow; 75:1 split ratio; 1 µL injection; oven method: 25 °C isotherm for 6 min, 10 °C/min to 60 °C, 20 °C/min to 300 °C. Retention times: heptane 2.05 min, 1-octene 4.45 min, 2-methyloctanal 11.34 min, *n*-nonanal 11.83 min, diphenyl ether 14.19 min.

Due to the large excess of 1-alkene, resulting in the broad tailing alkene peak, it was not possible to detect small amounts of isomerization products.

4.v. ICP-MS:

The rhodium concentration was determined by ICP-MS either in Edinburgh by Dr. Eades using a Perkin Elmer Elan 6100 DRC Quad and a Thermo-Finnegan Element 2, or in St Andrews by Mrs. Williamson using an Agilent 7500 series ICP-MS spectrometer. Samples were diluted with an internal standard and all data was corrected for metal content determined in the corresponding blank.

Aliquots of the catalyst solutions were treated with concentrated nitric acid and hydrogen peroxide solution (30 wt. %) (1 mL each). The solution was carefully evaporated to dryness, topped up with water, evaporated to dryness. It was quantitatively transferred to a 1 mL volumetric flask and topped up and analysed by ICP-MS as stated above.

Metal leaching experiments:

The organic layer from the hydroformylation reactions using each metalloenzyme were analysed by ICP-MS for Rh content at the end of the reaction. Thus, the values given represent the maximum build-up Rh in the organic phase throughout the reaction.

Table S11: Rhodium content in the organic phase after hydroformylation.

Entry	Metalloenzyme	Rh ppb	% Rh[b]	N[c]
**1**	SCP-2L A100C-**1**-**P1**-Rh	15.1 (± 1.4)	0.3 ± 0.0	2
**2**	SCP-2L A100C-**1**-**P2**-Rh	158.2 (± 112.2)	2.0 ± 2.0	3
**3**	SCP-2L A100C-**1**-**P3**-Rh	138.4 (± 68.7)	5.8 ± 2.9	6
**4**	SCP-2L V83CC-**1**-**P1**-Rh	42.7 (± 23.5)	1.4 ± 0.8	2

**5**	SCP-2L V83CC-**1**-**P2**-Rh	260.8 (± 15.1)	2.6 ± 1.3	5
**6**	SCP-2L V83C-**1**-**P3**-Rh	21.6 (± 12.2)	0.5 ± 0.3	6

a. Determined in organic phase by ICP-MS. b. Ratio of the Rh concentration in the organic phase after the reaction over the initial Rh concentration in the aqueous phase (as determined by ICP-MS). d. Number of experiments.

4.vi. Characterisation of protein after catalytic reaction:

After the catalytic reaction the mass of the protein in the aqueous layer was analysed (Figure S11). SCP-2L A100C-**1-P3(O)** was the main observed product, as one might expect from oxidation during work-up. The protein precipitate was dissolved in 8M urea, and mass spectrometry also showed SCP-2L A100C-**1-P3(O)** (Figure S12).

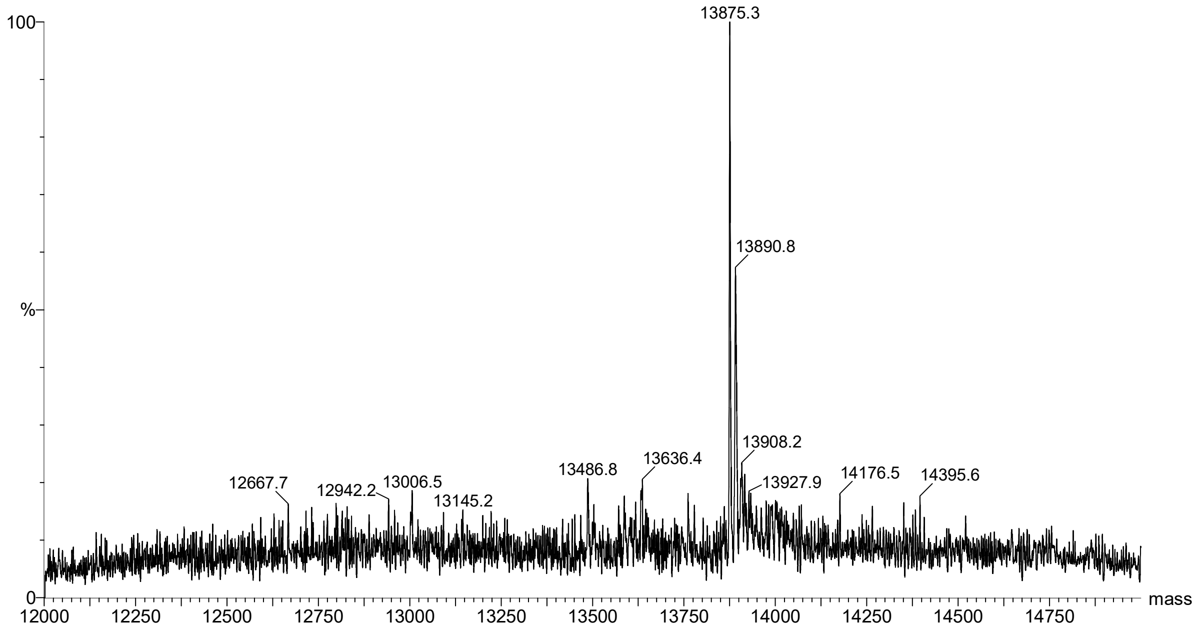

Figure S12: Protein MS after reaction – aqueous layer (Mass = Da)

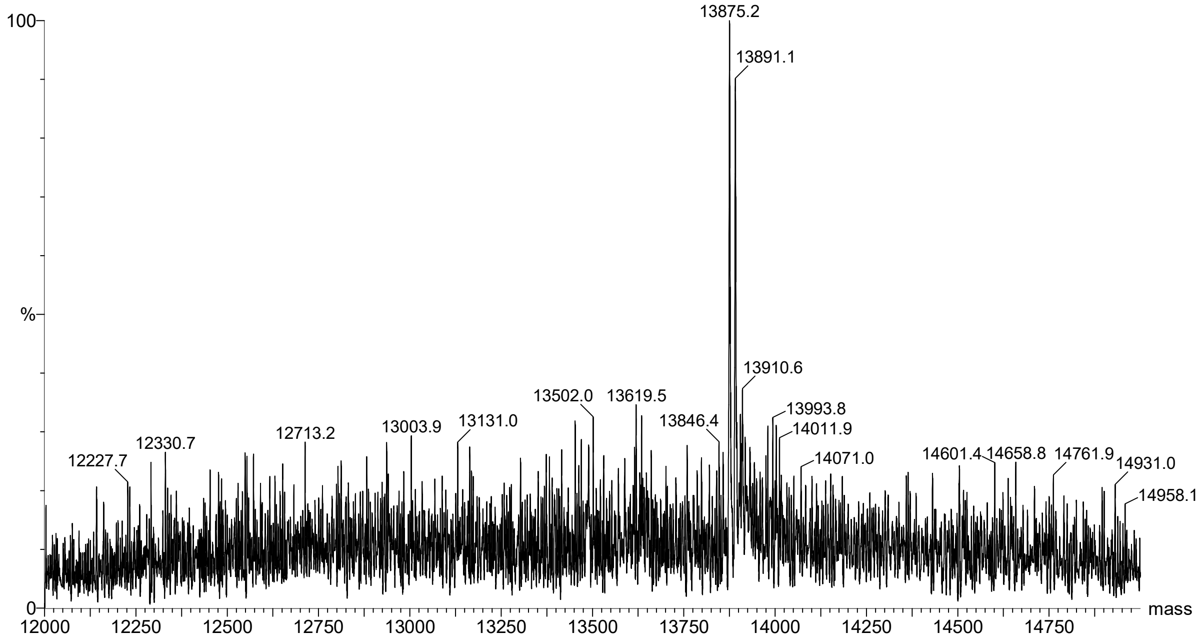

Figure S13: Protein MS after reaction – precipitate dissolved in 8M urea (Mass = Da)

5. Rate calculations

Comparing the rate of hydroformylation using SCP-2L A100C-**1**-**P3**-Rh against TPPTS would enable an indication to be made about the effect of the protein scaffold and if the resulting catalyst was acting like an enzyme in providing a significant rate enhancement. Simply comparing the TONs for the Rh:TPPTS catalysed reaction with our enzyme allows us to make a crude comparison. Using the data for the TPPTS reaction with 1:30 Rh:P ratio at 260 nmol Rh (the closest result in terms of selectivity) gives a rate enhancement in the order of 10^3 for 1-octene and 10^4 for 1-decene of the metalloproteins over Rh/TPPTS. This value is calculated from comparing the turnovers for 1-octene of the enzyme and Rh/TPPTS, 409 and 1.04 respectively. This shows the enzyme is approximately 400 times faster. However, this value does not take into account that the Rh loading in the enzyme is 11 times lower than in the Rh/TPPTS system (260 vs. 23 nmol). As the reaction rate of hydroformylation is first order in the concentration of Rh, this needs to be taken into account and leads to a rate enhancement of approximately 4000. The same approach was taken to estimate the rate enhancement for 1-decene.

However, it is not clear that this is the real rate enhancement. In biphasic systems, the presence of 'free Rh' increases the apparent reaction rate and decreases the selectivity. Even at 30 eq. of TPPTS the linearity is still only 72%, which is much lower than the highest reported in the literature - >95% at 1:80 Rh:P ratio, at 400 ppm Rh.[15] This could suggest that in the TPPTS controls we still have a small amount of 'free-Rh' in the organic alkene phase and that the actual TON in the aqueous phase would be even lower, meaning the above quoted rate-enhancements are an underestimate.

Using literature rate constants for the biphasic Rh-catalysed hydroformylation of 1-octene,[15] our enzyme rate enhancement can be estimated to be over one million.

Table S12: Literature rate constants for hydroformylation calculated for our reaction conditions.

Entry	T (°C)	P (bar)	[Rh] (ppm)	Alkene	Rate constant at 125 °C (min^{-1})[15]	Rate constant at 35 °C (min^{-1})	Rate constant at lower conc., A (min^{-1})
**1**	125	80	300-400	1-octene	4.6 x 10^{-4}	8.9 x 10^{-7}	1.5 x 10^{-8}
**2**	125	80	300-400	1-decene	1.50 x 10^{-4}	2.9 x10^{-7}	4.8 x 10^{-9}

To enable the literature data to be compared with our results it needs to be corrected for temperature and rhodium concentration. An estimate on the change in rate across temperature is that the rate of a reactions roughly doubles on increasing the temperature by 10 °C. Therefore, the rate at 35 °C as opposed to 125 °C can be obtained by dividing the rate by 2^9. Once again, the rate needs to be corrected for rhodium concentration. In the literature, the Rh concentration is reported as 300-400 ppm whilst in our experiments the Rh concentration is 5 ppm, therefore 60 times smaller. The corrected rate constant is given as **A** in Table S12. This rate constant can now be compared to the ArM data. To get a crude rate constant for the ArM the TON over 48 h can be converted to a rate **B**.

Table S13: Calculation of rate enhancement for the ArM system in hydroformylation.

Entry	Alkene	A	Estimated ArM rate constant, B (min^{-1})	Rate enhancement B/A
**1**	1-octene	1.5 x 10^{-8}	1.4 x 10^{-1}	9.5 x 10^{6}
**2**	1-decene	4.8 x 10^{-9}	4.7 x 10^{-2}	9.7 x 10^{6}

This shows we observe a rate enhancement of 9 x 10^6 for SCP-2L A100C-**1**-**P3**-Rh over the Rh/TPPTS system (Table S12).

References

[1] P. J. Deuss, G. Popa, C. H. Botting, W. Laan, P. C. J. Kamer, *Angew. Chem. Int . Ed.* **2010**, *49*, 5315-5317
[2] R. B.Kapust, J. Toezser, J. D. Fox, D. E. Anderson, S. Cherry, T. D. Copeland, D. S. Waugh, *Protein Eng.* **2001,** *14*, 993.
[3] H. Huanting, J. H. Naismith, *BMC Biotechnology* **2008**, *8*, 91.
[4] P. W. N. M. van Leeuwen, D. Rivillo, M. Raynal, Z. Freixa, *J. Am. Chem. Soc.* **2011**, *133,* 18562–18565.
[5] D. Rivillo, H. Gulyás, J. Benet-Buchholz, E. C. Escudero-Adán, Z. Freixa P. W. N. M. van Leeuwen, *Angew. Chem., Int. Ed. Engl.* **2007**, *46*, 7247–7250.
[6] M. M. Bradford, *Anal. Biochem.* **1976**, *72*, 248–254.
[7] P. J. Deuss, G. Popa, C. H. Botting, W. Laan, P. C. J. Kamer, *Angew. Chem., Int. Ed.* **2010**, *49*, 5315-5317.
[8] M. Newville, *J. Synchrot. Radiat.* **2001**, *8*, 322-324.
[9] B. Ravel, M. Newville, *J. Synchrot. Radiat.* **2005**, *12*, 537- 541.
[10[T. B. Dansen, J. Westerman, F. S. Wouters, R. J. A. Wanders, A. Van Hoek, T. W. J. Gadella, Jr., K. W. A. Wirtz *Biochem. J.* **1999**, *339*, 193-199.
[11] P. J. Deuss, G. Popa, A. M. Z. Slawin, W. Laan, P. C. J. Kamer, *ChemCatChem* **2013**, *5*, 1184-1191.
[12] H. M. Key, D. S. Clark, J. F. Hartwig, *J. Am. Chem. Soc.* **2015**, *137*, 8261-8268.
[13] R. K. Arvela, N. E. Leadbeater, M. S. Sangi, V. A. Williams, P. Granados, R. D. Singer, *J. Org. Chem.* **2005**, *70*, 161-168.
[14] *Rhodium Catalysed Hydroformylation* (Eds. P. W. N. M. Van Leeuwen, C. Claver) Kluwer Academic Publishers, New York, **2002**.
[15] B. Cornils, W. A. Herrmann, *Aqueous-Phase Organometallic Catalysis*, Wiley-VCH, Weiheim, **2004**, p. 394.

Author Contributions

P.C.J.K conceived and designed the project. W.L, P.J.D, L.O, and A.G.J prepared the metalloproteins and conducted catalytic testing. P.P.W, E.K.G and A.G.J designed and performed the EXAFS experiments. A.G.J. prepared the SeMet metalloproteins. P.P.W analysed the EXAFS data. A.G.J, P.J.D and P.C.J.K wrote the manuscript with input from all authors.
